# Characterisation of Variants of Cyclic di-GMP Turnover Proteins Associated with Semi-Constitutive rdar Morphotype Expression in Commensal and Uropathogenic *Escherichia coli* Strains

**DOI:** 10.3390/microorganisms11082048

**Published:** 2023-08-09

**Authors:** Annika Cimdins-Ahne, Ali-Oddin Naemi, Fengyang Li, Roger Simm, Ute Römling

**Affiliations:** 1 Department of Microbiology, Tumor and Cell Biology, Karolinska Institutet, 171 77 Stockholm, Sweden; a.cimdins@hotmail.de (A.C.-A.); fylee1987@outlook.com (F.L.); 2 Institute of Oral Biology, University of Oslo, 0313 Oslo, Norway; a.o.naemi@odont.uio.no (A.-O.N.); roger.simm@odont.uio.no (R.S.); 3 Norwegian Veterinary Institute, 0106 Oslo, Norway

**Keywords:** rdar morphotype, c-di-GMP, biofilm, protein variants, GGDEF, EAL

## Abstract

Expression of rdar (red, dry, and rough) colony morphology-based biofilm formation in *Escherichia coli* is highly variable. To investigate the molecular mechanisms of semi-constitutive rdar morphotype formation, we compared their cyclic di-GMP turnover protein content and variability to the highly regulated, temperature-dependent morphotype of the historical and modern ST10 isolates *E. coli* MG1655 and Fec10, respectively. Subsequently, we assessed the effects of cyclic di-GMP turnover protein variants of the EAL phosphodiesterases YcgG and YjcC and the horizontally transferred diguanylate cyclase DgcX on biofilm formation and motility. The two YcgG variants with truncations of the N-terminal CSS signaling domain were oppositely effective in targeting downregulation of rdar biofilm formation compared to the full-length reference protein. Expression of the C-terminal truncated variants YjcC_Fec67_ and YjcC_Tob1_ showed highly diminished apparent phosphodiesterase activity compared to the reference YjcC_MG1655_. For YjcC_Fec101_, substitution of the C-terminus led to an apparently inactive enzyme. Overexpression of the diguanylate cyclase DgcX contributed to upregulation of cellulose biosynthesis but not to elevated expression of the major biofilm regulator *csgD* in the “classical” rdar-expressing commensal strain *E. coli* Fec10. Thus, the c-di-GMP regulating network is highly complex with protein variants displaying substantially different apparent enzymatic activities.

## 1. Introduction

The genomic variability of organisms contributes to their adaptive potential. Microorganisms, particularly bacteria with their small genomes often in multiple copies, are masters of stress survival and adaptation. While a major focus has been on the effects of horizontally transferred accessory elements, which undoubtedly introduce novel features into bacterial genomes in quantum leaps, it has recently been realized that even single nucleotide polymorphisms (SNPs) can have a substantial effect on the cell’s physiology, metabolism, and virulence. These SNPs have been mainly explored upon being non-synonymous in open reading frames (ORFs). For example, non-synonymous mutations in a variety of ORFs have been shown to contribute to the enhanced virulence/reduced biofilm phenotype of the emerged African invasive ST313 clone of the gastrointestinal pathogen *Salmonella enterica* serovar Typhimurium [1]. However, few studies have also explored SNPs in the more variable intergenic regions and in promoter regions. For example, the insertion/transversion of a single nucleotide in the promoter region of the major rdar biofilm regulator CsgD promotes temperature and stress sigma factor RpoS-independent expression of the downstream *csgDEFG* operon [2].

Biofilm formation, the capability of microorganisms to build up multicellular communities supported by extracellular matrix, is an intrinsic feature of almost any bacterial species. The multifunctional biofilm can thereby play diverse determinative roles in the interaction with the environment equally, as with a eukaryotic host. The exopolysaccharide cellulose, a C6 phosphoethanolamine modified β-1,4-glucan with a partial crystalline structure, and depolymerization-inert amyloid curli fimbriae are the extracellular matrix components, the biosynthesis of which is triggered by the *csgD* rdar biofilm activator [3,4,5,6]. The resulting spore-like biofilm structure promotes long-term survival in the environment equally, as it restricts acute virulence in mammalian hosts [7]. Although *csgD* expression is a major regulatory hub from the transcriptional to the post-translational level, a major determinant of *csgD* expression and cellulose biosynthesis is the complex signaling network of cyclic di-GMP, a ubiquitous bacterial second messenger [8]. Several of the 31 *E. coli* and 22 *S. typhimurium* proteins with a GGDEF diguanylate cyclase, an EAL phosphodiesterase domain, or an evolved EAL domain devoid of catalytic activity and cyclic di-GMP binding regulate the expression of this central rdar biofilm activator [9,10]. Notably, SNPs in cyclic di-GMP turnover proteins have been demonstrated to determine biofilm expression and virulence [11].

We recently observed a high diversity of expression patterns of rdar biofilm formation among commensal and uropathogenic isolates of *E. coli* [12,13,14]. This variability contrasts with the almost uniform appearance of rdar morphotype expression in *S. typhimurium* and related serovars of *Salmonella enterica* [15].

Additionally, in *E. coli*, mutations in the promoter region of *csgD*, the major biofilm activator gene, have been found to promote semi-constitutive (including temperature-independent) rdar biofilm formation [2,16]. However, not all *E. coli* strains with semi-constitutive rdar biofilm formation possess promoter mutations; thus, other mechanisms must cause upregulation of *csgD* expression. As others, we observed high variability in the biofilm regulating the cyclic di-GMP network genes of *E. coli* strains. Usually, the effects of gene variants are not immediately accessible unless amino acids in conserved signature motifs required for catalytic activity are directly addressed by non-synonymous nucleotide substitutions or frameshifts, leading to a premature stop in the open reading frame. However, we recently noted that amino acid (aa) substitutions that occur outside of signature motifs determinative for catalytic activity can be crucial for apparent enzymatic activity and consequently may contribute to differential expression of the rdar colony biofilm [17]. In this context, distinct, single aa substitutions in the FI-PAS-GGDEF-EAL domain protein YciR, associated with upregulated rdar biofilm formation, reduced the regulatory activity at ambient and body temperatures in the commensal *E. coli* Tob1, chosen as the test strain.

As multiple components might contribute to the emergence of semi-constitutive rdar morphotype formation, in this work, we aimed to further unravel the molecular basis of semi-constitutive rdar biofilm expression in commensal and uropathogenic *E. coli* strains by comparative genomic analyses. We compared their genome sequences with those of ST10 *E. coli* strains, the commensal strain *E. coli* K-12 MG1655, and the recently isolated human commensal strain *E. coli* Fec10, which express a highly regulated rdar biofilm exclusively at ambient temperature [18]. With a focus on investigating the correlation between sequence variations of proteins involved in the turnover of the second messenger c-di-GMP and semi-constitutive rdar biofilm formation [19], we addressed the effects of alterations on the cyclic di-GMP phosphodiesterases YcgG and YjcC and the horizontal transfer of the diguanylate cyclase DgcX on semi-constitutive rdar biofilm expression. Our results show that two YcgG variants with several aa substitutions and slightly different truncations of the N-terminal signaling domain downregulated the semi-constitutive rdar biofilm to different degrees compared to the reference YcgG of *E. coli* MG1655. Equally, YjcC variants with aa substitutions and truncation/substitution of the C-terminal aa sequence displayed highly downregulated or abolished apparent catalytic activity. On the other hand, as overexpression of the diguanylate cyclase DgcX contributed to upregulation of cellulose biosynthesis but did not elevate production of the rdar biofilm regulator CsgD, additional components might contribute to the semi-constitutive rdar morphotype and *csgD* expression at 37 °C in the commensal strain *E. coli* Fec101.

## 2. Materials and Methods

### 2.1. Strains and Growth Conditions

All commensal and uropathogenic *E. coli* strains analyzed in this work have been previously described and genome sequenced, and the cyclic di-GMP turnover network has been characterized [14]. *E. coli* strains expressing the rdar colony morphotype semi-constitutively were *E. coli* Tob1, Fec67, Fec101, B8638, B-11870, 80//6, and No. 12. Strains were propagated on LB agar or grown on LB without salt plates supplemented with 2% Congo red (CR) and Coomassie brilliant blue G stock solution (CR at 2 mg/mL, Brilliant Blue G at 1 mg/mL in 70% (*v*/*v*) ethanol) as described to visualize the rdar biofilm phenotype [19]. If required, 100 µg/mL ampicillin and 0.1% L-arabinose were added to the medium for plasmid propagation and gene expression, respectively. All strains used in the study are listed in Table S1.

### 2.2. Cloning Procedures and Site-Directed Mutagenesis

Genes of interest were amplified and ligated into the pBAD30 vector [20] via XbaI/HindIII restriction sites. Site-directed mutagenesis was performed with phusion^®^ High-Fidelity DNA Polymerase and an overlap extension PCR approach using 17 cycles to introduce the mutations. A DpnI digest to remove methylated DNA ensured that only mutated plasmid was to be transformed. All constructs were verified by sequencing. All plasmids used in the study are listed in Table S2, and the primers are listed in Table S3.

### 2.3. Mutant Construction

Construction of the *dgcX* chromosomal deletion mutant was performed via λ red-mediated homologous recombination [21]. In brief, a kanamycin resistance cassette was amplified from the pKD4 template flanked by 40-bp homologous sequences at the beginning and end of the target gene. After electroporation into *E. coli* Fec101 carrying helper vector pKD46, the mutants were selected on 25 μg/mL and subsequently 50 μg/mL kanamycin, verified by PCR with primers flanking the replaced ORF and cured of pKD46 by incubation at 42 °C. Primers are listed in Table S3.

### 2.4. Analysis of rdar Colony Morphology and Type 1 Fimbriae Expression

Congo red LB without salt plates was used to visualize expression of the rdar morphotype. Dye binding to both cellulose and curli fibers stains the bacterial colony [2,9,12]. Strains were streaked for single colonies, or 5 μL of a suspension of OD_600_ = 5 was spotted onto the agar plates. If applicable, 100 µg/mL ampicillin and 0.1% L-arabinose were added. Development of the colony morphology and color was analyzed at 28 °C and 37 °C, respectively, and documented by taking photographs at distinct time points for up to 72 h. 

To visualize cellulose expression, 5 μL of a suspension of OD_600_ = 5 were spotted onto LB without salt agar plates supplemented with 50 μg/mL Calcofluor white (Fluorescent Brightener 28, Sigma, St. Louis, MO, USA). Fluorescence was observed under UV light of a wavelength of 365 nm.

Type 1 fimbriae were assessed by the pellicle assay performed in standing culture in LB medium at 37 °C for 48 h. Pellicles were documented by taking photographs from above and as a side view.

### 2.5. Flagella-Based Motility

Strains were inoculated into LB motility plates containing 0.25% agar using a toothpick. If applicable, 100 µg/mL ampicillin and 0.1% L-arabinose were added. Agar plates were incubated at 28 °C and 37 °C for 6 h.

### 2.6. SDS PAGE and Western Blotting

To investigate CsgD production, the strains of interest were grown on LB without salt agar plates for 16–18 h. For the analysis of YcgG synthesis, strains were grown on LB without salt agar plates supplemented with 100 µg/mL ampicillin and 0.1% L-arabinose for 16 h. Five milligrams of cells were harvested and suspended in 1× SDS sample buffer, followed by boiling at 95 °C for 10 min. The samples were separated on a denaturing SDS PAGE (4% stacking and 12% (for detection of His-tagged YcgG) or 15% (for CsgD detection) resolving gel). Separated proteins in gels were visualized with a staining solution (0.1% Coomassie Brilliant Blue G-250, 2% (*w*/*v*) ortho-phosphoric acid, 10% (*w*/*v*) ammonium sulfate, (Sigma)) to allow for adjustment of equal protein content, or semidry western blotting was performed at 120 mA for 1 h to transfer the contained proteins onto a PVDF membrane (Immobilon P; Millipore, Burlington, MA, USA). CsgD protein was detected using a custom-made primary rabbit polyclonal anti-*E. coli* CsgD peptide antibody [22] and a goat anti-rabbit secondary antibody (Jackson Immuno Research) (both antibodies were diluted 1:3000). His-tagged proteins were detected using 1:1500 diluted mouse anti-Penta-His antibody (Qiagen, Hilden, Germany) and a 1:2000 diluted goat anti-mouse secondary antibody (Jackson Immuno Research). After treatment with Lumi-Light Western Blotting substrate (Roche, Basel, Switzerland), the resulting chemiluminescence was detected via an LAS-1000 detector (Fujifilm, Tokyo, Japan).

### 2.7. Bioinformatic Tools

The RAST-Server (Rapid Annotation using Subsystem Technology) was used for initial genome and proteome comparisons [23,24,25]. Prediction of functional protein domains was performed using SMART (accessed latest 8 June 2023, http://smart.embl-heidelberg.de) [26,27]. BLAST was used to retrieve homologous proteins [28]. The tool ClustalX 2.1 [29] or MUSCLE (http://www.ebi.ac.uk/Tools/msa/muscle/; [30]), which is implemented in AliView (version 1.17.1, [31]), was used to perform multiple sequence alignment. The Sequence editor http://www.fr33.net/seqedit.php, the Expasy translate tool http://web.expasy.org/translate/, and GeneDoc were used to view and manage sequences. ESPript software, version 3.0, was used to visualize sequence alignments [32]. Structural models were either taken from UniProt (Alphafold models) [33] or constructed with Phyre2 [34]. Structural models were compared and visualized with Chimera software, version 1.14 [35]. Ident and Sim (www.bioinformatics.org/sms2/ident_sim.html; accessed latest 15 June 2023) were used to calculate the pairwise identity and similarity of aligned protein sequences.

### 2.8. Analyzed Protein Sequences

The YjcC sequences aligned in Figure 3 were (clockwise) YjcC_ECOL6, A0A0H2VD18_ECOL6 of *E. coli* CFT073; YjcC_ECO27, B7UPN0_ECO27 of EPEC strain E2348/69; YjcC_ECO8N, A0A0H3ESW7_ECO8N of AIEC strain NRG 857C; YjcC_ECO45, B7MJS8_ECO45 of ExPEC strain S88; YjcC_ECOK1, A0A0H2Z591_ECOK1 of APEC O1:K1; YjcC_ECO57, A0A0H3JK63_ECO57 of O157:H7; YjcC_ECOBD, A0A140NEW3_ECOBD of strain B BL21-DE3; YjcC_ECOLI, P32701 of *E. coli* K-12; YjcC_ECOHS, A0A7I6HB99_ECOHS of strain HS; YjcC_ECO24, A7ZUT3_ECO24 of ETEC strain E24377A; YjcC_ECO55, B7LB02_ECO55 of EAEC strain 55989; YjcC_SHISS, Q3YUS4_SHISS of *Shigella sonnei* Ss046; YjcC_CVM_N17EC1334, EAC1547616.1; YjcC_BLSE174, HCL8611405.1; YjcC_FSIS11705879, EFA9343023.1; YjcC_33, HBN0449758.1; and YjcC_EPECa14, EFZ42063.1.

The GGDEF domains investigated in Figure 4 were from the proteins (clockwise direction) *Shigella sonnei* EGF2695153.1 and EFZ2348867.1, *E. coli* WP_00058633.1, *E. coli* TW14182 WP_000293658.1, *Salmonella kentucky* EBV8389552.1, *Escherichia albertii* WP_105197891.1, *Escherichia* WP_105281683.1, *Citrobacter* sp. R56 WP_203358054.1, *Escherichia fergusonii* KWW06214.1, *E. fergusonii* WP_002432239.1, *E. albertii* KAF0954534.1, *Escherichia* WP_123058197.1, *Escherichia* WP_165382775.1, *Klebsiella pneumoniae* WP_134392503.1, *E. fergusonii* HAJ6531924.1, *Enterobacter hormaechei* subsp. *xiangfangensis* MBT1749060.1, and *Enterobacter ludwigii* WP_063214496.1.

## 3. Results

We identified two additional candidate genes encoding cyclic di-GMP turnover proteins associated with semi-constitutive rdar morphotype expression, the function of which is potentially altered/modulated by non-synonymous single nucleotide changes in combination with 5′ or 3′ alteration/truncation of the ORFs. These genes were *ycgG* coding for a redox-regulated cyclic di-GMP phosphodiesterase, previously shown to be involved in regulation of motility [36]; and *yjcC* encoding a phosphodiesterase (PDE), previously shown to mediate rdar morphotype regulation in *E. coli* [37,38]. In addition, *dgcX* coding for a horizontally transferred diguanylate cyclase (DGC) first identified in the 2001 EAEC outbreak strain [39] has been identified in the commensal strain Fec101.

### 3.1. The N-Terminal Sequence of Truncated YcgG Affects Protein Expression and rdar Morphotype Formation

**Figure 1:**
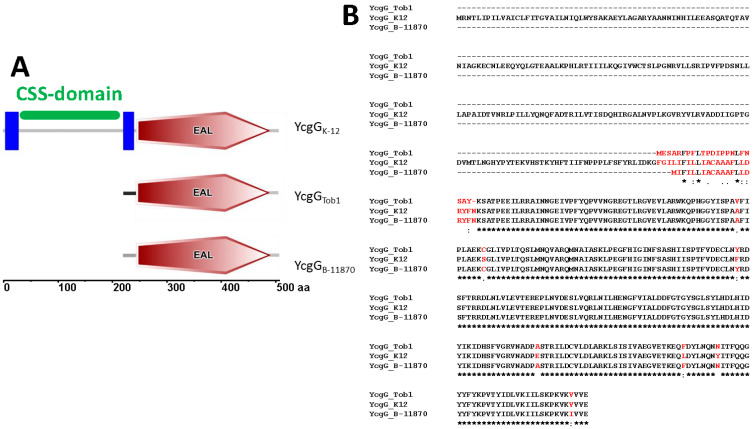
YcgG is truncated in commensal and uropathogenic *E. coli* strains. (**A**) Domain organization of YcgG from *E. coli* K-12 (MG1655 and Fec10), commensal Tob1, and uropathogenic B-11870 as depicted by SMART server (accessed latest 8 June 2023, http://smart.embl-heidelberg.de/). Tob1 and B-11870 lack the N-terminal CSS signaling domain (indicated in green) compared to K-12. The N-terminal region of truncated YcgG is unique to *E. coli* Tob1 (black line). (**B**) Alignment of aa sequences of YcgG from *E. coli* K-12, Tob1, and B-11870. YcgG proteins from Tob1 and B-11870 are truncated at the N-terminus compared to the K-12 reference protein. The alignment was created with MUSCLE (http://www.ebi.ac.uk/Tools/msa/muscle/). Amino acid substitutions are indicated in red letters. Star * indicates conservation of amino acid, : indicates conservative substitution; blank indicates non-conservative substitution; . indicates semi-conservative substitution.

In *E. coli* K-12 MG1655, the PDE YcgG consists of an N-terminal redox responsive CSS domain of around 200 aas [40,41] flanked by transmembrane domains and a C-terminal EAL domain. A full-length YcgG protein is present also in the modern equivalent of *E. coli* K-12. the commensal strain Fec10, in the commensal strain Fec101, and in the uropathogenic strain B-8638. *E. coli* Fec101 and B8628 both express a semi-constitutive rdar morphotype. As previously reported, YcgG from Fec10 and Fec101 possesses the aa substitution Y474N, while YcgG of B-8638 contains six additional aa substitutions [17]. In contrast, in the commensal strains Tob1 and Fec67 and the uropathogenic strains B-11870 (including the clonal variant 80//6) and No.12, YcgG has a truncated N-terminus, leaving basically only the EAL domain. Notably, the truncated nucleotide regions are not entirely identical, resulting in YcgG from *E. coli* Tob1 to possess a two aa longer and divergent 22 aa stretch at the N-terminus compared to the truncated YcgG variant of YcgG_B-11870_, where the N-terminus is congruent with the aa sequence of YcgG_MG1655_ at that position (Figure 1). Further, YcgG_Tob1_ and YcgG_B-11870_ contain aa substitutions compared to YcgG_MG1655_ (Figure 1). The aa exchange A298V is unique to YcgG_Tob1_; S306C, F358Y, E437A, L467F, and Y474N substitutions are present in YcgG_Tob1_, and YcgG_B-11870_ and V504I are unique to YcgG_B-11870_ (Figure 1, [17]). Furthermore, the ORF of *ycgG_B-11870_* has the rare start codon TTG in contrast to *ycgG_MG1655_* and *ycgG_Tob1_*, which have ATG as a start codon. However, changing TTG to the conventional ATG start codon has recently been demonstrated not to substantially alter protein expression in *E. coli* [42].

**Figure 2:**
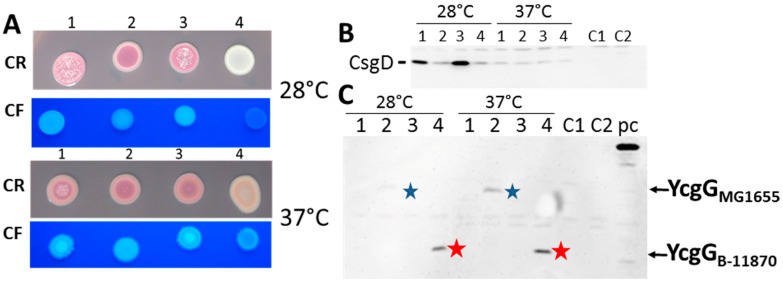
Differential activity of YcgG from *E. coli* Tob1, B-11870, and MG1655 ST10 strains. (**A**) Biofilm formation on LB without salt agar plates supplemented with Congo red or Calcofluor white after 48 h of incubation at 28 °C or 37 °C. (**B**) Western blot detection of CsgD from cells grown on LB agar plates for 16 h at 28 °C or 37 °C. CsgD was detected with an anti-CsgD antibody as described previously [43,44]. (**C**) Western blot detection of 6xHis-tagged YcgG from cells grown on LB agar plates without salt for 16 h at 28 °C or 37 °C. Recombinant YcgG was detected with an anti-Penta-His antibody (Qiagen). 1 = Tob1 (pBAD30), 2 = Tob1 (pYcgG_MG1655_-6xHis), 3 = Tob1 (pYcgG_Tob1_-6xHis), 4 = Tob1 (pYcgG_B-11870_-6xHis). C1 = Tob1 ∆*csgD* incubated at 28 °C for 16 h, C2 = Tob1 ∆*csgD* incubated at 37 °C for 16 h. All plates supplemented with 100 µg/mL ampicillin and 0.1% L-arabinose. pc = ClpG-6xHis of *Pseudomonas aeruginosa* SG17M. Blue and red stars indicate the signals detected for YcgG_MG1655_ and YcgG_B-11870_, respectively.

To investigate the consequences of the expression of the highly similar, yet distinct proteins with deletion of the CSS sensory domain, we analyzed the effect of expression of YcgG_MG1655_, YcgG_Tob1_, and YcgG_B-11870_ on rdar morphotype formation and production of its major activator CsgD in the semi-constitutive rdar strain *E. coli* Tob1 (rdar_28 °C_/rdar_37 °C_) at 28 °C and 37 °C (Figure 2A). To restrict the assessment of functionality solely on the gene product, the coding regions were cloned under the control of the L-arabinose-inducible pBAD promoter in pBAD30 with an identical Shine–Dalgarno sequence. While full-length YcgG_MG1655_ slightly downregulated rdar morphotype formation and the protein level of the master rdar biofilm regulator CsgD, expression of truncated YcgG_B-11870_ unexpectedly led to a highly reduced rdar morphotype and low levels of CsgD production (Figure 2B). In contrast, expression of YcgG_Tob1_ was less effective than YcgG_MG1655_ in downregulating the rdar colony morphotype and had no visible effect on CsgD synthesis. Notably, the effect of YcgG production on rdar morphotype formation was consistently observed at 28 °C and 37 °C. CsgD levels could only be reliably assessed at 28 °C. Although expression of soluble protein at 37 °C is at the detection limit of western blot, we judged that none of the constructs affected CsgD production at 37 °C (Figure 2B; [17]).

To investigate whether downregulation of rdar biofilm formation and its major activator is due to altered protein production levels or impaired enzymatic activity of YcgG upon truncation, we detected the production of YcgG via the C-terminal 6xHis-tag by western blotting. While production of YcgG_MG1655_ wild-type protein was detected at 28 °C and 37 °C, YcgG_B-11870_ was produced to a significantly greater extent. Notably, production of YcgG_B-11870_ was increased at 37 °C, suggesting that elevated temperature or decreased proteolysis stabilizes this YcgG variant. Concomitant with the high production of YcgG_B-11870_, a specific non-biofilm forming, smooth, and white (saw) morphotype emerged at 28 °C. In contrast, production of YcgG_Tob1_ could not be observed by western blot analysis despite visible effects on rdar colony morphology (Figure 2C). In summary, the effect on rdar morphotype formation and CsgD expression of the YcgG variants strongly correlates with their protein production levels.

### 3.2. YjcC Proteins with C-Terminal and Individual Amino Acid Substitutions Show Diminished Apparent Phosphodiesterase Activity and Downregulation of the rdar Colony Morphotype

**Figure 3:**
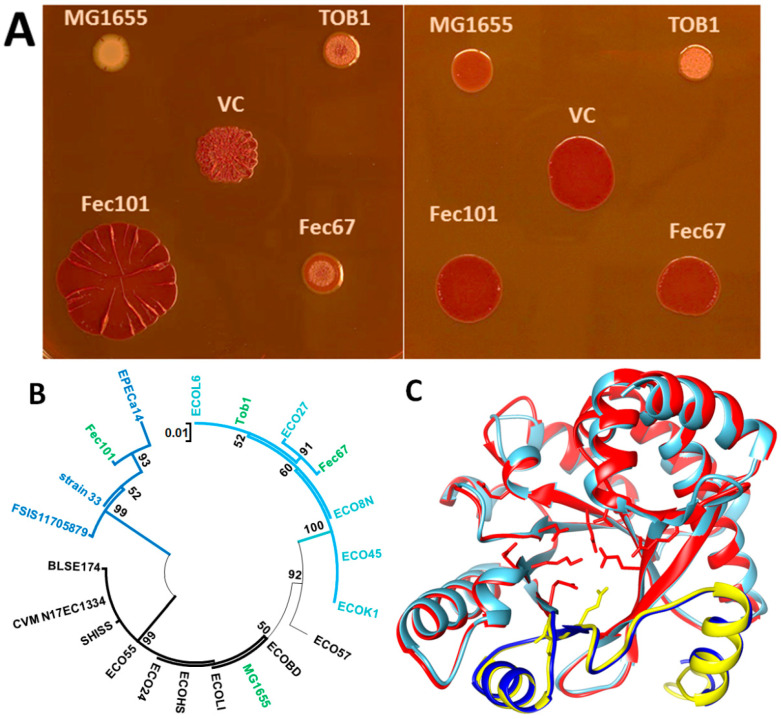
Characterization of YjcC variants of semi-constitutive *E. coli* isolates compared to YjcC of *E. coli* K-12 MG1655. (**A**) Variants of *yjcC* from *E. coli* Tob1, Fec67, Fec101, and K-12 MG1655 were cloned into pBAD30 using an identical Shine–Dalgarno sequences and an extended region downstream of the stop codon. The constructs were expressed in *E. coli* Tob1. VC = vector control pBAD30. LB without salt plates supplemented with 100 µg/mL ampicillin, 0.1% arabinose, and Congo red incubated at 28 °C (**left** panel) and 37 °C (**right** panel) for 48 h and 24 h, respectively. (**B**) Phylogenetic tree of YjcC variants of investigated *E. coli* strains MG1655, Tob1, Fec67, and Fec101 [17] and additional variants retrieved by Blast search from NCBI and UniProt databases (Section 2 [28,33]). Alignment was conducted with ClustalX 2.1, and phylogenetic relationships were assessed with the maximum-likelihood approach using all sites and 1000 bootstrap replications. YjcC (by strain name) subgroups are indicated by different branch colors. (**C**) Overlaid Phyre2 structural models of the EAL domains of YjcC_MG1655_ (red) and YjcC_Fec101_ (blue). Diverging C-terminal sequences are in yellow (YjcC_MG1655_) and dark blue (YjcC_Fec101_). Amino acids involved in catalysis, substrate, and divalent ion binding and loop six stabilization are indicated with their side chains. Functional aas with yellow side chains not present in YjcC_Fec101_ are involved in divalent cation binding.

The phosphodiesterase YjcC is a major phosphodiesterase in *S. typhimurium*, determinative of temperature-dependent rdar colony morphotype expression [37]. In *E. coli*, MG1655 *yjcC* appears to play a more restricted role with alternative phosphodiesterases, being dominant with respect to affecting rdar biofilm colony morphology [38]. We noted variants of the phosphodiesterase YjcC in the semi-constitutive rdar morphotype-expressing strains *E. coli* Tob1, Fec101, and Fec67 (Figure S3). In strains Tob1 and Fec67, the C to T transition at position 1552 of the *yjcC* ORF created a stop codon leading to a 11 aa shorter protein (Figure S2). In strain Fec101, the deletion of an adenosine nucleotide at position 1433 led to a frameshift and the substitution of the last 49 C-terminal aa of YjcC with a 44 aa long unrelated sequence (Figure S3). In addition, YjcC proteins of strains Tob1 and Fec67 possess multiple, partly distinct, aa substitutions compared to reference YjcC_MG1655_, while YjcC_Fec101_ displays two aa substitutions (P309L D403E) (Figure S3; [17]). To investigate the impact of the YjcC variants with the altered C-termini, we cloned the ORFs under the control of the same Shine–Dalgarno sequence and with the same extension of the nucleotide sequences 3′ of the stop codon (Table S1). Expression of these constructs and reference YjcC_MG1655_ in *E. coli* Tob1 showed production of YjcC_MG1655_ to substantially downregulate the rdar colony morphotype at 28 °C (Figure 3A). While the partially truncated protein variant YjcC_Tob1_ and, to a minor extent, YjcC_Fec67_ still downregulated the rdar morphotype at 28 °C, YjcC_Fec101_ did not substantially alter the rdar colony morphology type compared to the vector control. Notably, YjcC_MG1655_ and YjcC_Fec67_ expression downregulated the rdar morphotype also at 37 °C to a minor extent, while expression of YjcC_Tob1_ displayed a substantially altered, although still downregulated, rdar colony morphology. We therefore must assume that the two aa substitutions that discriminate YjcC_Fec67_ from YjcC_Tob1_ are responsible for this different effect on the rdar colony morphotype.

### 3.3. YjcC Proteins with Similar C-Terminal and Amino Acid Substitutions Are Present in the Database

The YjcC phosphodiesterase proteins with C-terminal substitutions and truncations are not unique to our strain collection (Figure S2). YjcC proteins with the 11 aa shorter C-terminus and the same or similar aa substitutions, such as YjcC_Tob1_/YjcC_Fec67_, compared to YjcC_MG1655_ are found encoded by a number of isolates among the sequenced *E. coli* strains including the uropathogenic strain *E. coli* CFT073. Equally, at least eight human *E. coli* isolates, including a human commensal, pathogens such as strain EPECa14, animal, and wastewater strains, possess a YjcC with a C-terminal substituted aa sequence highly similar to YjcC_Fec101_ with the second glutamate of the EVTE motif involved in divalent cation binding missing (Figure S2). Notably, distinct single nucleotide deletions and deletion of several nucleotides led to a similar C-terminal aa sequence substitution in YjcC in these epidemiologically unrelated strains. The phylogenetic tree clearly discriminates among these three distinct classes of YjcC protein variants (Figure 3B). Despite a divergent C-terminus, however, the structural model indicates a similar structure of the C-terminus of YjcC_Fec101_ compared to YjcC_MG1655_ (Figure 3C).

### 3.4. The Novel Diguanylate Cyclase DgcX Is Critical for Biofilm Formation at Human Body Temperature

**Figure 4:**
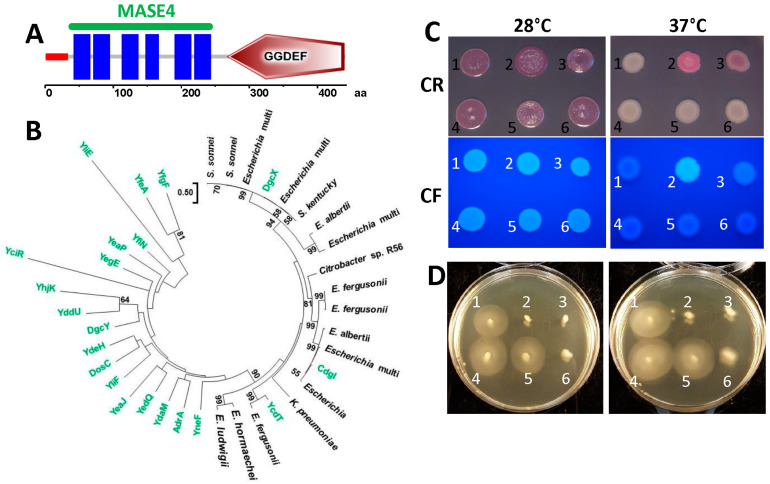
DgcX is an additional diguanylate cyclase horizontally transferred into *E. coli* Fec101. (**A**) Domain organization of DgcX by the SMART server. (**B**) Phylogenetic tree of the GGDEF domains of GGDEF and GGDEF-EAL domain proteins of *E. coli* ST10 [17] compared to the GGDEF domains of the newly introduced DgcX and selected proteins with high aa identity belonging to the MASE4-GGDEF family. In *E. coli*, the MASE4-GGDEF family includes the functional diguanylate cyclases DgcX and YcdT (DgcT) and the catalytically inactive protein YeaI (CdgI) as described in the text. (**C**) Colony morphology of *E. coli* Fec10 (rdar_28 °C_/saw_37 °C_) expressing DgcX or catalytic mutants, grown for 24 h on Congo-red (upper panel) or Calcofluor white (lower panel) agar plates at 28 °C and 37 °C. 1 = Fec10 VC, 2 = pDgcX_N_, 3 = pDgcX_C_, 4 = pDgcX_E360A_, 5 = pDgcX_E359A_, 6 = pDgcX_E359A, E360A_. VC = pBAD30. (**D**) Swimming motility of *E. coli* Fec10 (rdar_28 °C_/saw_37 °C_) expressing DgcX or catalytic mutants, grown for 6 h on LB swimming plates at 28 °C and 37 °C. 1. pBAD30, 2. pDgcX_N_, 3. p:DgcX_C_, 4. p gcX_E359A,E360A_, 5. pDgcX_E360A_, 6. pDgcX_E359A_. (**C**,**D**): Plates supplemented with 100 µg/mL ampicillin and 0.1% arabinose. pDgcX = *dgcX* cloned in pBAD30.

The DGC DgcX, not present in *E. coli* K-12 and Fec10, was first described as a highly expressed diguanylate cyclase in the *E. coli* O104:H4 2011 German outbreak strain, and it is considered to be a virulence determinant (Figure 4A; [39]). The genome of the commensal *E. coli* strain Fec101 showed only minor deviations in the c-di-GMP network and the biofilm components compared to the ST10 reference strains with respect to SNPs and gene rearrangements, but it contains the additional DGC DgcX, a MASE4-GGDEF domain protein [17,39,44]. DgcX was thus identified as a candidate diguanylate cyclase responsible for the observed upregulated rdar colony morphotype at 37 °C in the commensal strain Fec101.

Interestingly, the chromosomal location and gene context of *dgcX* vary among the strains harboring this gene. Database searches reveal that the O104:H4 outbreak strain and other EAEC strains harbor *dgcX* and the adjacent inserted prophage between *ybhC* (position complement 805998–807281 relative to the *E. coli* K-12 MG1655 genome) and *ybhB* (position complement 807433–807909). The nature of the prophage as such differs, however [39]. In the environmental strain *E. coli* SE11, *dgcX*, along with an adjacent prophage, is inserted between *ydaW* (position 1422701–1423200) and *uspF* (position complement 1435185–1435619) [39]. In Fec101, *dgcX* is present as part of a 9-kb insertion yet at another location between *argW* and *dsdC* (Figure S3A). In *E. coli* K-12 MG1655, the prophage CPS-53/KpLE1 [45] is inserted between *argW* (position 2466309–2466383 in K-12 MG1655) and *dsdC* (position complement 2476694–2477629).

### 3.5. Overexpression of DgcX Induces Cellulose Production at 37 °C in the Regulated rdar Colony Morphology Type Strain E. coli Fec10

DgcX harbors a GGDEF domain that is seemingly functional as a diguanylate cyclase, with an intact GGEEF catalytic motif and other conserved aa motifs required for catalytic activity, substrate binding, and metal ion coordination [46,47]. N-terminal of the GGDEF domain, DgcX contains a MASE4 domain [35] with six transmembrane regions (Figure 4A). The closest homologs to DgcX over the entire length of the protein in *E. coli* K-12 is the catalytically non-functional YeaI (CdgI) and the diguanylate cyclase YcdT (Figure 4B and Figure S3). Compared to other DGCs of *E. coli*, DgcX is highly expressed at both 28 °C and 37 °C [35].

To test the effect of DgcX against the Fec101 background, we constructed a *dgcX* deletion mutant (Figure S3B). Assessment of rdar morphotype expression, however, showed only a minor effect of the *dgcX* deletion on rdar biofilm formation, while the deletion mutant of the gene for the major biofilm activator *csgD* displayed a residual pink, dry, and rough (pdar) morphotype indicating cellulose but no curli fiber biosynthesis.

**Figure 5:**
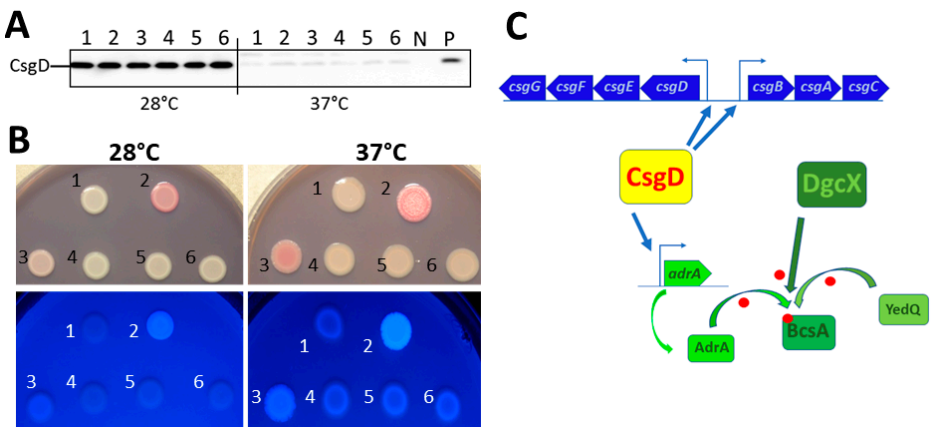
DgcX stimulates cellulose biosynthesis but not *csgD* expression. (**A**) Western blot detection of CsgD in *E. coli* Fec10 harboring plasmids expressing wild-type and catalytic mutants of DgcX. N = negative control *E. coli* Tob2 (Tob1 Δ*csgD*; pBAD30), P = positive control *E. coli* Tob1 (pBAD30). (**B**) rdar morphotype of *E. coli* Fec10 ∆*csgD* (saw_28 °C_/saw_37 °C_) harboring plasmids expressing wild-type and catalytic mutants of DgcX were grown on Congo red or Calcofluor white plates for 24 h at 28 °C and 37 °C. (**A**,**B**): 1 = Fec10 VC, 2 = pDgcX_N_, 3 = pDgcX_C_, 4 = pDgcX_E360A_, 5 = pDgcX_E359A_, 6 = pDgcX_E359A, E360A_. pDgcX = *dgcX* cloned in pBAD30. VC = pBAD30. Plates were supplemented with 100 µg/mL ampicillin and 0.1% arabinose. (**C**) Model regulation of cellulose biosynthesis by different genetic factors. CsgD activates curli expression directly and provides a positive feedback loop on its own expression in *E. coli* [49]. Cellulose biosynthesis is indirectly activated by *csgD* via expression of the DGC AdrA. However, expression of the DGC DgcX, equally as the DGC YdeQ, can stimulate cellulose expression independently of *csgD* at both 28 °C and 37 °C.

To investigate the effect of *dgcX* overexpression on rdar morphotype formation, *dgcX* was cloned with either an N- or C-terminal 6xHis-tag into the cloning vector pBAD30 under the control of an L-arabinose-controlled promoter. DgcX was subsequently expressed in strain *E. coli* Fec10, which features a highly regulated rdar morphotype at 28 °C and a saw morphotype at 37 °C, which facilitates assessment of the functionality of a diguanylate cyclase [12,18]. Overexpression of *dgcX* enhanced the rdar morphotype at 28 °C (Figure 4C, left panel) and resulted in a pdar morphotype and enhanced Calcofluor white binding at 37 °C (Figure 4C, right panel), suggesting *csgD*-independent cellulose production. Upregulation of cellulose expression is dependent on the catalytic activity, as DgcX variants with mutations of the GGEEF motif to GGEAF or GGAAF no longer induced upregulation of rdar morphotype expression, while modest upregulation in colony morphology was still observed in the GGAEF mutant. Remarkably, overexpression of DgcX did not alter production of CsgD in Fec10 at either 28 °C or 37 °C (Figure 5A). This outcome is in contrast to observations in the 2011 *E. coli* outbreak strain, in which DgcX elevated CsgD production, but cellulose was not synthesized due to a frame shift mutation in the gene encoding the c-di-GMP binding protein BcsE [39]. BcsE has recently been shown to be required for optimal cellulose production in *E. coli* and *S. typhimurium* [48]. Moreover, to clearly show the effect of DgcX at 28 °C, we expressed DgcX in the Fec10 ∆*csgD* strain. Expression of *dgcX* induced pdar morphotype expression also at 28 °C but to a lesser extent (Figure 5B). Thus, DgcX conditionally contributes to semi-constitutive rdar morphotype expression in Fec101 by upregulation of *csgD*-independent cellulose expression.

Notably, the effect of DgcX on cellulose expression is remarkably stronger for the N-terminally, rather than for the C-terminally, tagged construct with barely visible activity at 28 °C indicating that the tag either interferes with (at the C-terminus) or promotes (at the N-terminus) enzymatic activity and/or protein stability (Figure 4C and Figure 5B).

### 3.6. Effect of dgcX on Flagella-Based Motility in E. coli Fec101

Cyclic di-GMP signaling regulates the lifestyle transition from sessility (biofilm formation) to motility. We were therefore wondering whether *dgcX* affects motility. We have previously shown that Fec101 exhibits moderate motility [17]. The corresponding motility assay showed that *E. coli* Fec101 Δ*dgcX* has slightly higher motility than the Fec101 wild type at 28 °C and 37 °C (Figure S3B).

Consistent with the motility phenotype of the *dgcX* deletion mutant, overexpression of *dgcX* in Fec101 repressed motility at 28 °C and 37 °C. As expected with the predicted loss of the diguanylate cyclase activity, the GGAAF and GGEAF mutants did not repress motility, while the GGAEF mutant still displayed motility repression, suggesting, as in the case of regulation of rdar colony morphology, at least residual catalytic activity of the DgcX GGAEF variant (Figure 4D). Thus, in DgcX, unexpectedly, the second glutamate of the GGEEF motif is dominant in determining catalytic activity [46,47].

### 3.7. DgcX Is Restricted to E. coli Species

Previously, *dgcX* was found to be encoded by the genomes of the EAEC strains LB226692, 2011C-3493, HUSEC041, 55989, 2009EL-2071, 2009EL-2050, ETEC H10407, E24377A, and the commensal strain SE11 [39,44]. A protein BLAST search against the NCBI microbial genomes database revealed the presence of DgcX (>75% query cover and >50% identity of the aa sequence) in more than 1150 strains of the genus *Escherichia*, mostly *E. coli*, a few *Shigella* spp., and *Escherichia albertii* (Figure 4B and BLAST search performed in June 2023). Thus, the presence of *dgcX*, although obtained through horizontal gene transfer, is indeed restricted to *E. coli*. In conclusion, the gene product DgcX is highly conserved and present only in strains of *E. coli* and closely related species of the *Escherichia* genus, while the highly similar gene products YcdT and YeaI/CdgI can also be present in related genera (Figure 4B and Figure S3C,D). This outcome suggests recent diversifying radiation of the YcdT/DgcX/YeaI family specifically in *E. coli* with one of its members, DgcX, subject to extended mobilization. Identity/similarity analyses of the aligned sequences showed, however, that threshold identity/similarity values can be determined to categorize proteins of the YcdT/DgcX/YeaI subfamilies, in line with above-mentioned threshold values with the potential assignment of founders for novel protein subfamilies. (Figure S3C,D).

## 4. Discussion

Within a species, biofilm formation can be highly variable; however, the underlying molecular mechanisms in natural isolates remain to be determined. We have started to dissect the molecular basis of the temperature-independent rdar colony morphotype expression of three commensal and three uropathogenic *E. coli* strains compared to the commensal strain *E. coli* MG1655 and its recently isolated ‘modern’ ST10 counterpart *E. coli* Fec10. Both of these strains express a highly regulated rdar biofilm only at temperatures less than 30 °C [17,18]. These analyses subsequently revealed high variability in the enzymes that conduct turnover of the second messenger c-di-GMP associated with differential biofilm expression. In a subset of strains, distinct aa substitutions in the trigger diguanylate cyclase/phosphodiesterase YciR were experimentally coupled with reduced ability to downregulate *csgD* encoding the master regulator of rdar biofilm formation and, upon introduction of a nonsense mutation, even to upregulate *csgD* by a truncated FI-PAS-GGDEF protein acting as an apparent diguanylate cyclase [17]. However, additional alterations in alternative cyclic di-GMP turnover proteins might cumulatively contribute to semi-constitutive rdar morphotype expression in *E. coli* strains. In this work, we investigated the effect of genomic alterations occurring in *ycgG* and *yjcC* coding for redox-regulated cyclic di-GMP phosphodiesterases and the effect of the acquisition of *dgcX*, which codes for a horizontally transferred diguanylate cyclase in the commensal strain *E. coli* Fec101. DgcX was first identified in the 2011 EAEC outbreak strain [39]. Findings in this work could be extended to investigate the molecular basis of substantially different behaviors of protein variants equally as natural evolution toward potentially novel protein functionalities in their physiological and evolutionary contexts. For example, the specific feature of *yjcC* in regulating the temperature dependence of the rdar morphotype in *S. typhimurium* in contrast to *E. coli* representatives might be an acquired virulence trait specific for either *Salmonella* or *E. coli*, causing intestinal infection.

Although the EAL domain of the CSS-EAL protein YcgG is present in all investigated *E. coli* strains, the N-terminal part of the protein was truncated in a subset of isolates due to deletion of larger genomic sequences. Indeed, we have also observed a deletion of the CSS domain of YcgG in all strains of the multidrug-resistant pandemic *E. coli* ST131 clone (unpublished observations). However, the deletion of the N-terminal periplasmic CSS sensory domain left in all cases an ORF encoding an intact EAL phosphodiesterase domain. The deletions, however, also led to distinct protein variants that, upon overexpression, showed opposite performance in production levels and consequently downregulated the rdar morphotype relative to the ST10 *E. coli* wild-type protein. Thereby only the effect of the *ycgG_Tob1_* variant resulting in a lower protein level is in congruence with the expression of a semi-constitutive rdar morphotype. It is possible that the rare TTG start codon of *ycgG*_Tob1_ causes significantly lower protein production. Alternatively, the novel unique 22-aa sequence at the truncated N-terminus and/or, less likely, the distinct nucleotide substitution A298V can affect protein folding, stability, or degradation, causing YcgG_Tob1_ to be undetectable by standard western blot analyses, although its effect is still visible in the biological assay. However, it remains to be determined how *ycgG* expression has been altered upon introduction of the novel promoter in vivo in *E. coli* strain Tob1 (Figure S1D). Although expression of *ycgG* is almost undetectable in *E. coli* K-12 [36,50], deletion of a truncated *ycgG* (c1610), similar to *ycgG_B-11870_*, in the *E. coli* UPEC strain CFT073 increased the levels of the FimA subunit of type 1 fimbriae and enhanced adherence to bladder epithelial cells [51]. Thus, although we did not observe a decrease in type 1 fimbriae-dependent biofilm formation of *E. coli* Tob1 upon overexpression of the *ycgG* variants in laboratory assays (Figure S4), truncated *ycgG_B-11870_* might decrease biofilm formation in other strains and/or under alternative conditions, such as in the host. In this context, *ycgG* in neonatal meningitis-causing *E. coli* (NMEC) and the uropathogenic isolate UTI89 is involved in the switch from iron acquisition to citrate fermentation via the citrate transporter CitT [52]. Whether the truncation of YcgG is causative for involvement in this metabolic activity has not been investigated.

In summary, the effect of *ycgG* variants is only partially congruent with enhanced rdar biofilm formation and might predominantly be involved in the regulation of alternative cyclic di-GMP-affected physiological and metabolic properties. YcgG belongs to a group of five redox sensing CSS-EAL domain proteins in *E. coli* [40,41]. Reducing conditions or deletion of the disulfide bond formation system components led to proteolytic cleavage of the YjcC and YlaB proteins, which resulted in reduced catalytic activity of the cytoplasmic EAL-only domain [41]. It is intriguing that the manifested genomic alterations observed here for *ycgG* in natural commensal and uropathogenic *E. coli* strains, alternative transcriptional start sites and additive proteolytic digest by the periplasmic proteases DegQ and DegP in the model strain *E. coli* K-12 under laboratory growth conditions led to a similar fragmentation pattern of a CSS-EAL protein [14,17,41,49,51,52,53].

**Figure 6:**
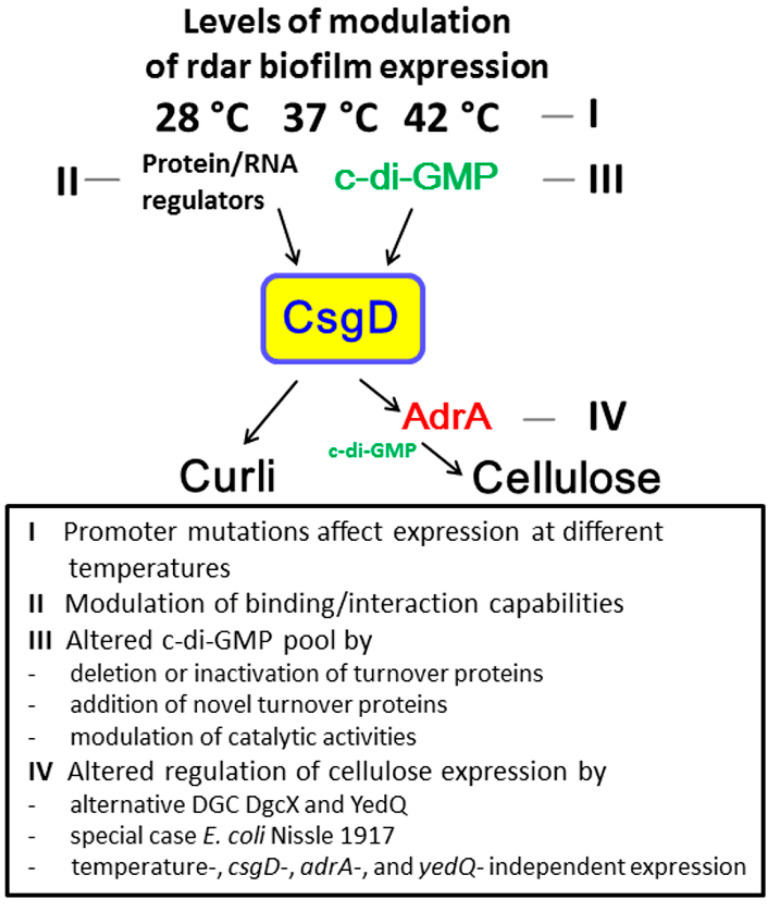
Different modes of regulation of rdar biofilm expression by genetic and environmental factors. Modes of regulating rdar morphotype formation can be at the transcriptional level by mutations in the *csgD* promoter, leading to altered expression at 28 °C, 37 °C, and 42 °C (I) [2,16], by different genetic factors including global transcriptional regulators and small RNAs targeting the *csgD* promoter and beyond (II); by alterations of cyclic di-GMP turnover protein activity and presence, such as altered catalytic activity, including phosphodiesterase and diguanylate cyclase activity; or by deletion and acquisition of novel diguanylate cyclases (III) [10,38,39]. Cyclic di-GMP is a major determinative factor for overriding most other regulatory determinants. CsgD subsequently activates curli expression directly. Cellulose biosynthesis can be regulated independently of *csgD*-mediated rdar biofilm expression (IV) [4,8,54].

In theory, at least three fundamentally different modes of regulation of semi-constitutive rdar biofilm expression by cyclic di-GMP turnover proteins can occur: reduced (apparent) phosphodiesterase activity, upregulated diguanylate cyclase activity of a chromosomally encoded diguanylate cyclase, and acquisition of a novel diguanylate cyclase (Figure 6). In the commensal strain *E. coli* Fec101, the situation might be more complex. The GGDEF-EAL domain protein variant YciR still substantially reduces rdar biofilm formation [17], and as shown in this work, the horizontally introduced diguanylate cyclase DgcX does not enhance production of the biofilm activator CsgD but contributes to rdar biofilm formation by upregulation of cellulose biosynthesis. Indeed, diguanylate cyclases transcriptionally independent of *csgD* have been shown to exclusively regulate cellulose biosynthesis in *E. coli* under alternative growth conditions [54]. The dedication of a diguanylate cyclase/phosphodiesterase to preferentially affect biofilm formation, a particular biofilm component, or motility is not uncommon.

In *S. typhimurium*, YjcC is the dominant phosphodiesterase-mediating temperature-dependent rdar morphotype [37]. In *E. coli* MG1655, *yjcC* has a less determinative role [38]. The investigated *yjcC* variants *yjcC_Tob1_*, *yjcC_Fec67_*, and *yjcC_Fec101_* showed subsequently decreased activity. Although YjcC_Fec101_ seems to be catalytically inactive (as it lacks a glutamate required for divalent cation binding), it remains to be shown whether lack of this phosphodiesterase activity would be sufficient to promote CsgD production in Fec101 at 37 °C. On the other hand, the substitution of the last 49 C-terminal aa with a 44 aa-long unrelated sequence in YjcC_Fec101_ might have led to a protein with a novel functionality that is not immediately visible by substantial alterations in the rdar colony morphology biofilm type. However, the substantially different appearance of the colony morphology of *E. coli* Tob1 upon overexpression of *yjcC_Fec101_* at 28 °C indicates expression and a physiological role of *yjcC_Fec101_*.

## 5. Conclusions

In conclusion, characterization of the effects of variants of cyclic di-GMP specific phosphodiesterases on the downregulation of rdar colony morphotype expression and regulation of motility provided indications for their contributions to the semi-constitutive rdar morphotype, which is seen at high frequency in *E. coli* strains compared to the closely related species *S. typhimurium*.

## Data Availability

Not applicable.

## Supplementary Materials

The following supporting information can be downloaded at: https://www.mdpi.com/article/10.3390/microorganisms11082048/s1, Figure S1: Nucleotide sequence of the *ycgG* region from *E. coli* K-12 MG1655, commensal Tob1, and uropathogenic B-11870; Figure S2: Multiple alignment of YjcC of *E. coli* strains MG1655, Tob1, Fec67, and Fec101; Figure S3: Flanking region of the DGC encoding gene *dgcX* in Fec101; Figure S4: Pellicle assay indicative for the expression of type 1 fimbriae. Table S1: Strains constructed or used in this study; Table S2: Plasmids constructed or used in this study; Table S3: Oligonucleotides used in this study. References [55,56] are cited in the supplementary materials as reference [6] and [7], respectively.

## References

[1] Sokaribo A.S., Balezantis L.R., MacKenzie K.D., Wang Y., Palmer M.B., Chung B., Herman N.J., McCarthy M.C., Chen J.M., White A.P. (2021). A SNP in the cache 1 signaling domain of diguanylate cyclase STM1987 leads to increased in vivo fitness of invasive *Salmonella* strains. Infect. Immun..

[2] Römling U., Sierralta W.D., Eriksson K., Normark S. (1998). Multicellular and aggregative behaviour of *Salmonella typhimurium* strains is controlled by mutations in the *agfD* promoter. Mol. Microbiol..

[3] Thongsomboon W., Serra D.O., Possling A., Hadjineophytou C., Hengge R., Cegelski L. (2018). Phosphoethanolamine cellulose: A naturally produced chemically modified cellulose. Science.

[4] Zogaj X., Nimtz M., Rohde M., Bokranz W., Römling U. (2001). The multicellular morphotypes of *Salmonella typhimurium* and *Escherichia coli* produce cellulose as the second component of the extracellular matrix. Mol. Microbiol..

[5] Chapman M.R., Robinson L.S., Pinkner J.S., Roth R., Heuser J., Hammar M., Normark S., Hultgren S.J. (2002). Role of *Escherichia coli* curli operons in directing amyloid fiber formation. Science.

[6] Hammar M., Arnqvist A., Bian Z., Olsén A., Normark S. (1995). Expression of two *csg* operons is required for production of fibronectin- and congo red-binding curli polymers in *Escherichia coli* K-12. Mol. Microbiol..

[7] Lamprokostopoulou A., Römling U. (2022). Yin and Yang of biofilm formation and cyclic di-GMP signaling of the gastrointestinal pathogen *Salmonella enterica* Serovar Typhimurium. J. Innate Immun..

[8] Römling U., Galperin M.Y., Gomelsky M. (2013). Cyclic di-GMP: The first 25 years of a universal bacterial second messenger. Microbiol. Mol. Biol. Rev..

[9] Ahmad I., Cimdins A., Beske T., Römling U. (2017). Detailed analysis of c-di-GMP mediated regulation of *csgD* expression in *Salmonella typhimurium*. BMC Microbiol..

[10] Sarenko O., Klauck G., Wilke F.M., Pfiffer V., Richter A.M., Herbst S., Kaever V., Hengge R. (2017). More than enzymes that make or break cyclic di-GMP-local signaling in the interactome of GGDEF/EAL domain proteins of *Escherichia coli*. mBio.

[11] Römling U., Cao L.-Y., Bai F. (2023). Evolution of cyclic di-GMP signaling on a short and long term time scale. Microbiology.

[12] Bokranz W., Wang X., Tschäpe H., Römling U. (2005). Expression of cellulose and curli fimbriae by *Escherichia coli* isolated from the gastrointestinal tract. J. Med. Microbiol..

[13] Kai-Larsen Y., Lüthje P., Chromek M., Peters V., Wang X., Holm A., Kadas L., Hedlund K.O., Johansson J., Chapman M.R. (2010). Uropathogenic *Escherichia coli* modulates immune responses and its curli fimbriae interact with the antimicrobial peptide LL-37. PLoS Pathog..

[14] Cimdins A., Lüthje P., Li F., Ahmad I., Brauner A., Römling U. (2017). Draft genome sequences of semiconstitutive red, dry, and rough biofilm-forming commensal and uropathogenic *Escherichia coli* isolates. Genome Announc..

[15] Römling U., Bokranz W., Rabsch W., Zogaj X., Nimtz M., Tschäpe H. (2003). Occurrence and regulation of the multicellular morphotype in *Salmonella* serovars important in human disease. Int. J. Med. Microbiol..

[16] Uhlich G.A., Keen J.E., Elder R.O. (2001). Mutations in the *csgD* promoter associated with variations in curli expression in certain strains of *Escherichia coli* O157:H7. Appl. Environ. Microbiol..

[17] Cimdins A., Simm R., Li F., Lüthje P., Thorell K., Sjöling A., Brauner A., Römling U. (2017). Alterations of c-di-GMP turnover proteins modulate semi-constitutive rdar biofilm formation in commensal and uropathogenic *Escherichia coli*. Microbiologyopen.

[18] Kamal S.M., Cimdins-Ahne A., Lee C., Li F., Martin-Rodriguez A.J., Seferbekova Z., Afasizhev R., Wami H.T., Katikaridis P., Meins L. (2021). A recently isolated human commensal *Escherichia coli* ST10 clone member mediates enhanced thermotolerance and tetrathionate respiration on a P1 phage-derived IncY plasmid. Mol. Microbiol..

[19] Römling U. (2001). Genetic and phenotypic analysis of multicellular behavior in *Salmonella typhimurium*. Methods Enzymol..

[20] Guzman L.M., Belin D., Carson M.J., Beckwith J. (1995). Tight regulation, modulation, and high-level expression by vectors containing the arabinose PBAD promoter. J. Bacteriol..

[21] Datsenko K.A., Wanner B.L. (2000). One-step inactivation of chromosomal genes in *Escherichia coli* K-12 using PCR products. Proc. Natl. Acad. Sci. USA.

[22] Monteiro C., Saxena I., Wang X., Kader A., Bokranz W., Simm R., Nobles D., Chromek M., Brauner A., Brown R.M. (2009). Characterization of cellulose production in *Escherichia coli* Nissle 1917 and its biological consequences. Environ. Microbiol..

[23] Aziz R.K., Bartels D., Best A.A., DeJongh M., Disz T., Edwards R.A., Formsma K., Gerdes S., Glass E.M., Kubal M. (2008). The RAST Server: Rapid annotations using subsystems technology. BMC Genom..

[24] Brettin T., Davis J.J., Disz T., Edwards R.A., Gerdes S., Olsen G.J., Olson R., Overbeek R., Parrello B., Pusch G.D. (2015). RASTtk: A modular and extensible implementation of the RAST algorithm for building custom annotation pipelines and annotating batches of genomes. Sci. Rep..

[25] Overbeek R., Olson R., Pusch G.D., Olsen G.J., Davis J.J., Disz T., Edwards R.A., Gerdes S., Parrello B., Shukla M. (2014). The SEED and the Rapid Annotation of microbial genomes using Subsystems Technology (RAST). Nucleic Acids Res..

[26] Letunic I., Doerks T., Bork P. (2015). SMART: Recent updates, new developments and status in 2015. Nucleic Acids Res..

[27] Schultz J., Milpetz F., Bork P., Ponting C.P. (1998). SMART, a simple modular architecture research tool: Identification of signaling domains. Proc. Natl. Acad. Sci. USA.

[28] Altschul S.F., Gish W., Miller W., Myers E.W., Lipman D.J. (1990). Basic local alignment search tool. J. Mol. Biol..

[29] Higgins D.G., Sharp P.M. (1988). CLUSTAL: A package for performing multiple sequence alignment on a microcomputer. Gene.

[30] Edgar R.C. (2004). MUSCLE: Multiple sequence alignment with high accuracy and high throughput. Nucleic Acids Res..

[31] Larsson A. (2014). AliView: A fast and lightweight alignment viewer and editor for large datasets. Bioinformatics.

[32] Robert X., Gouet P. (2014). Deciphering key features in protein structures with the new ENDscript server. Nucleic Acids Res..

[33] Apweiler R., Bairoch A., Wu C.H., Barker W.C., Boeckmann B., Ferro S., Gasteiger E., Huang H., Lopez R., Magrane M. (2004). UniProt: The Universal Protein knowledgebase. Nucleic Acids Res..

[34] Kelley L.A., Mezulis S., Yates C.M., Wass M.N., Sternberg M.J. (2015). The Phyre2 web portal for protein modeling, prediction and analysis. Nat. Protoc..

[35] Pettersen E.F., Goddard T.D., Huang C.C., Couch G.S., Greenblatt D.M., Meng E.C., Ferrin T.E. (2004). UCSF Chimera—A visualization system for exploratory research and analysis. J. Comput. Chem..

[36] Reinders A., Hee C.S., Ozaki S., Mazur A., Boehm A., Schirmer T., Jenal U. (2016). Expression and genetic activation of cyclic di-GMP-specific phosphodiesterases in *Escherichia coli*. J. Bacteriol..

[37] Simm R., Lusch A., Kader A., Andersson M., Römling U. (2007). Role of EAL-containing proteins in multicellular behavior of *Salmonella enterica* serovar Typhimurium. J. Bacteriol..

[38] Sommerfeldt N., Possling A., Becker G., Pesavento C., Tschowri N., Hengge R. (2009). Gene expression patterns and differential input into curli fimbriae regulation of all GGDEF/EAL domain proteins in *Escherichia coli*. Microbiology.

[39] Richter A.M., Povolotsky T.L., Wieler L.H., Hengge R. (2014). Cyclic-di-GMP signalling and biofilm-related properties of the Shiga toxin-producing 2011 German outbreak *Escherichia coli* O104:H4. EMBO Mol. Med..

[40] Römling U. (2005). Characterization of the rdar morphotype, a multicellular behaviour in Enterobacteriaceae. Cell. Mol. Life Sci..

[41] Herbst S., Lorkowski M., Sarenko O., Nguyen T.K.L., Jaenicke T., Hengge R. (2018). Transmembrane redox control and proteolysis of PdeC, a novel type of c-di-GMP phosphodiesterase. EMBO J..

[42] Himeno H., Masaki H., Kawai T., Ohta T., Kumagai I., Miura K., Watanabe K. (1987). Unusual genetic codes and a novel gene structure for tRNA(AGYSer) in starfish mitochondrial DNA. Gene.

[43] Römling U., Rohde M., Olsen A., Normark S., Reinköster J. (2000). AgfD, the checkpoint of multicellular and aggregative behaviour in *Salmonella typhimurium* regulates at least two independent pathways. Mol. Microbiol..

[44] Povolotsky T.L., Hengge R. (2016). Genome-based comparison of cyclic di-GMP signaling in pathogenic and commensal *Escherichia coli* strains. J. Bacteriol..

[45] Rudd K.E. (1999). Novel intergenic repeats of *Escherichia coli* K-12. Res. Microbiol..

[46] Schirmer T. (2016). C-di-GMP Synthesis: Structural Aspects of Evolution, Catalysis and Regulation. J. Mol. Biol..

[47] Römling U., Liang Z.X., Dow J.M. (2017). Progress in understanding the molecular basis underlying functional diversification of cyclic dinucleotide turnover proteins. J. Bacteriol..

[48] Fang X., Ahmad I., Blanka A., Schottkowski M., Cimdins A., Galperin M.Y., Römling U., Gomelsky M. (2014). GIL, a new c-di-GMP-binding protein domain involved in regulation of cellulose synthesis in enterobacteria. Mol. Microbiol..

[49] Hufnagel D.A., Evans M.L., Greene S.E., Pinkner J.S., Hultgren S.J., Chapman M.R. (2016). The Catabolite Repressor Protein-Cyclic AMP Complex Regulates *csgD* and Biofilm Formation in Uropathogenic *Escherichia coli*. J. Bacteriol..

[50] Weber H., Pesavento C., Possling A., Tischendorf G., Hengge R. (2006). Cyclic-di-GMP-mediated signalling within the sigma network of *Escherichia coli*. Mol. Microbiol..

[51] Spurbeck R.R., Tarrien R.J., Mobley H.L. (2012). Enzymatically active and inactive phosphodiesterases and diguanylate cyclases are involved in regulation of motility or sessility in *Escherichia coli* CFT073. mBio.

[52] Zlatkov N., Uhlin B.E. (2019). Absence of Global Stress Regulation in *Escherichia coli* promotes pathoadaptation and novel c-di-GMP-dependent metabolic capability. Sci. Rep..

[53] Thomason M.K., Bischler T., Eisenbart S.K., Förstner K.U., Zhang A., Herbig A., Nieselt K., Sharma C.M., Storz G. (2015). Global transcriptional start site mapping using differential RNA sequencing reveals novel antisense RNAs in *Escherichia coli*. J. Bacteriol..

[54] Bernal-Bayard J., Gomez-Valero L., Wessel A., Khanna V., Bouchier C., Ghigo J.M. (2018). Short genome report of cellulose-producing commensal *Escherichia coli* 1094. Stand. Genom. Sci..

[55] Rochon M., Römling U. (2006). Flagellin in combination with curli fimbriae elicits an immune response in the gastrointestinal epithelial cell line HT-29. Microbes Infect..

[56] Lee C., Franke K.B., Kamal S.M., Kim H., Lünsdorf H., Jäger J., Nimtz M., Trček J., Jänsch L., Bukau B. (2018). Stand-alone ClpG disaggregase confers superior heat tolerance to bacteria. Proc. Natl. Acad. Sci. USA.

