# Supplementary material for: Characterisation of Variants of Cyclic di-GMP Turnover Proteins Associated with Semi-Constitutive rdar Morphotype Expression in Commensal and Uropathogenic Escherichia coli Strains
Source: Microorganisms. 2023 Aug 9;11(8):2048. doi: 10.3390/microorganisms11082048 (PMC10459773; doi:10.3390/microorganisms11082048)
Supplement: Supplementary file 1 [file microorganisms-11-02048-s001.zip › microorganisms-2535656-supplementary.pdf]

microorganisms MDPI

Supplementary data

Characterisation of variants of cyclic di-GMP turnover proteins associated with semi-constitutive rdar morphotype expression in commensal and uropathogenic *Escherichia coli* strains

Annika Cimdins-Ahne [1], Ali-Oddin Naemi [2], Fengyang Li [1,#,] Roger Simm [2,3] and Ute Römling [1,*]

[1] Department of Microbiology, Tumor and Cell Biology, Karolinska Institutet, Stockholm, Sweden
[2] Institute of Oral Biology, University of Oslo, Oslo, Norway
[3] Norwegian Veterinary Institute, Oslo, Norway

# Current Affiliation: State Key Laboratory for Zoonotic Diseases, Key Laboratory of Zoonosis Research, Ministry of Education, College of Veterinary Medicine, Jilin University, Changchun, China

*Correspondence: ute.romling@ki.se

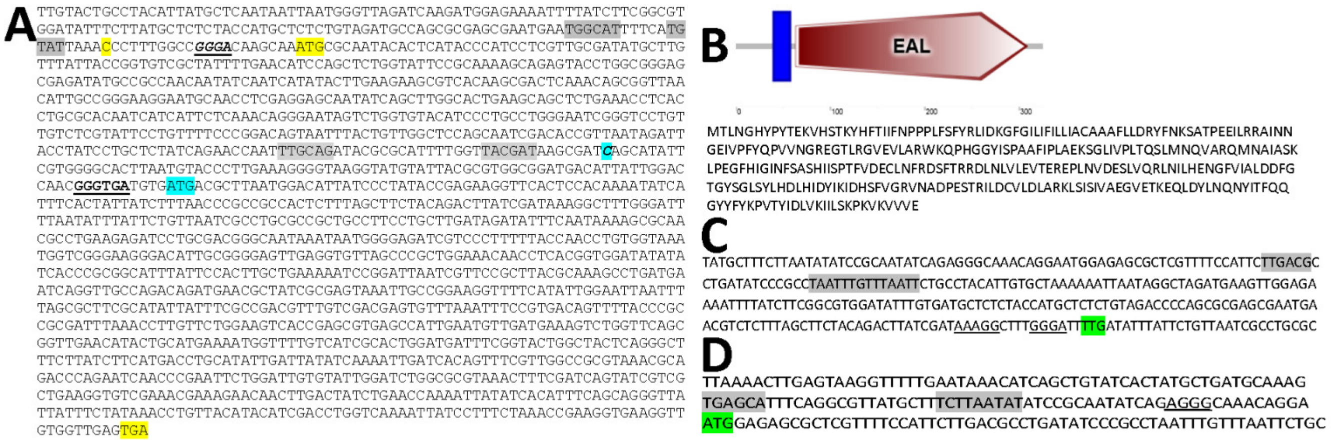

Figure S1. Nucleotide sequence of the *ycgG* region from *E. coli* K-12 MG1655, commensal Tob1, and uropathogenic B-11870. (**A**) Nucleotide sequence of the *E. coli* K-12 *ycgG* region; yellow marks the annotated start and stop codons as well as the putative transcriptional start site. The putative SD sequence is underlined upstream of the ATG start codon. The predicted sigma 54 promoter is marked in grey. An alternative transcriptional start site identified by RNA sequencing [43] is shown in blue. A putative promoter for this alternative start site is marked in grey and a putative SD sequence upstream of the alternative start codon is underlined. (**B**) Domain organization by the SMART server and amino acid sequence of the alternative gene product of the *ycgG* open reading frame. (**C**) Start codon (green) and predicted SD sequence (underlined) and upstream promoter (grey) for B-11870 *ycgG*. (**D**) Start codon (green), predicted SD sequence (underlined) and upstream promoter (grey) for *ycgG* from *E. coli* Tob1.

A

```
YjcC_EPECa14          MSHRARHQLLALPGIIFLVLFPIILSLWIAFLWAKSEVNNQLRTFAQLALDKSELVIRQA 60
YjcC_Fec101           MSHRARHQLLALPGIIFLVLFPIILSLWIAFLWAKSEVNNQLRTFAQLALDKSELVIRQA 60
YjcC_FSIS11705879     MSHRARHQLLALPGIIFLVLFPIILSLWIAFLWAKSEVNNQLRTFAQLALDKSELVIRQA 60
YjcC_33               MSHRARHQLLALPGIIFLVLFPIILSLWIAFLWAKSEVNNQLRTFAQLALDKSELVIRQA 60
YjcC_MG1655           MSHRARHQLLALPGIIFLVLFPIILSLWIAFLWAKSEVNNQLRTFAQLALDKSELVIRQA 60
YjcC_Fec67            MSHRARHQLLAFSGIIFLVLFPIILSLWIAFFWAKSEVNNQLRTFAQLALDKSELVIRQA 60
YjcC_Tob1             MSHRARHQLLAFPGIIFLVLFPIIMSLWIAFFWAKSEVNNQLRTFAQLALDKSELVIRQA 60
YjcC_CVM_N17EC1334    MSHRARHQLLALPGIIFLVLFPIILSLWIAFLWAKSEVNNQLRTFAQLALDKSELVIRQA 60
YjcC_BLSE174          MSHRARHQLLALPGIIFLVLFPIILSLWIAFLWAKSEVNNQLRTFAQLALDKSELVIRQA 60
                      ***********:.***********:******:****************************

YjcC_EPECa14          DLVSDAAERYQGQVCTPAHQKRMLNIIRGYLYINELIYARDNHFLCSSLIAPVNGYTIAP 120
YjcC_Fec101           DLVSDAAERYQGQVCTPAHQKRMLNIIRGYLYINELIYARDNHFLCSSLIAPVNGYTIAP 120
YjcC_FSIS11705879     DLVSDAAERYQGQVCTPAHQKRMLNIIRGYLYINELIYARDNHFLCSSLIAPVNGYTIAP 120
YjcC_33               DLVSDAAERYQGQVCTPAHQKRMLNIIRGYLYINELIYARDNHFLCSSLIAPVNGYTIAP 120
YjcC_MG1655           DLVSDAAERYQGQVCTPAHQKRMLNIIRGYLYINELIYARDNHFLCSSLIAPVNGYTIAP 120
YjcC_Fec67            DLVSDAAERYQGQVCTPAHQKRMLNIIRGYLYINELIYARDNHFLCSSLIASVNGYTIAP 120
YjcC_Tob1             DLVSDAAERYQGQVCTPAHQKRMLNIIRGYLYINELIYARDNHFLCSSLIASVNGYTIAP 120
YjcC_CVM_N17EC1334    DLVSDAAERYQGQVCTPAHQKRMLNIIRGYLYINELIYARDNHFLCSSLIAPVNGYTIAP 120
YjcC_BLSE174          DLVSDAAERYQGQVCTPAHQKRMLNIIRGYLYINELIYARDNHFLCSSLIAPVNGYTIAP 120
                      ***************************************************.********

YjcC_EPECa14          ADYKREPNVSIYYYRDTPFFSGYKMTYMQRGNYVAVINPLFWSEVMSDDPTLQWGVYDTV 180
YjcC_Fec101           ADYKREPNVSIYYYRDTPFFSGYKMTYMQRGNYVAVINPLFWSEVMSDDPTLQWGVYDTV 180
YjcC_FSIS11705879     ADYKREPNVSIYYYRDTPFFSGYKMTYMQRGNYVAVINPLFWSEVMSDDPTLQWGVYDTV 180
YjcC_33               ADYKREPNVSIYYYRDTPFFSGYKMTYMQRGNYVAVINPLFWSEVMSDDPTLQWGVYDTV 180
YjcC_MG1655           ADYKREPNVSIYYYRDTPFFSGYKMTYMQRGNYVAVINPLFWSEVMSDDPTLQWGVYDTV 180
YjcC_Fec67            ADYKREPNVSIYYYRDTPFFSGYKMTYMQRGNYVAVINPLFWSEVMSDDPTLQWGVYDTV 180
YjcC_Tob1             ADYKREPNVSIYYYRDTPFFSGYKMTYMQRGNYVAVINPLFWSEVMSDDPTLQWGVYDTV 180
YjcC_CVM_N17EC1334    ADYKREPNVSIYYYRDTPFFSGYKMTYMQRGNYVAVINPLFWSEVMSDDPTLQWGVYDTV 180
YjcC_BLSE174          ADYKREPNVSIYYYRDTPFFSGYKMTYMQRGNYVAVINPLFWSEVMSDDPTLQWGVYDTV 180
                      ************************************************************

YjcC_EPECa14          TKTFFSLSKEASAATFSPLIHLKDLTVQRNGYLYATVYSTKRPIAAIVATSYQRLITHFY 240
YjcC_Fec101           TKTFFSLSKEASAATFSPLIHLKDLTVQRNGYLYATVYSTKRPIAAIVATSYQRLITHFY 240
YjcC_FSIS11705879     TKTFFSLSKEASAATFSPLIHLKDLTVQRNGYLYATVYSTKRPIAAIVATSYQRLITHFY 240
YjcC_33               TKTFFSLSKEASAATFSPLIHLKDLTVQRNGYLYATVYSTKRPIAAIVATSYQRLITHFY 240
YjcC_MG1655           TKTFFSLSKEASAATFSPLIHLKDLTVQRNGYLYATVYSTKRPIAAIVATSYQRLITHFY 240
YjcC_Fec67            TKTFFSLSNEASAATFSPLIHLNDLTVQRNGYLYATVYSTKRPIAAIVATSYQRLITHFY 240
YjcC_Tob1             TKTFFSLSNEASAATFSPLIHLNDLTVQRNGYLYATVYSTKRPIAAIVATSYQRLITHFY 240
YjcC_CVM_N17EC1334    TKTFFSLSKEASAATFSPLIHLKDLTVQRNGYLYATVYSTKRPIAAIVATSYQRLITHFY 240
YjcC_BLSE174          TKTFFSLSKEASAATFSPLIHLKDLTVQRNGYLYATVYSTKRPIAAIVATSYQRLITHFY 240
                      ********:*************:*************************************

YjcC_EPECa14          NHLIFALPAGILGSLVLLLLWLRIRQNYLSPKRKLQRALEKHQLCLYYQPIIDIKTEKCI 300
YjcC_Fec101           NHLIFALPAGILGSLVLLLLWLRIRQNYLSPKRKLQRALEKHQLCLYYQPIIDIKTEKCI 300
YjcC_FSIS11705879     NHLIFALPAGILGSLVLLLLWLRIRQNYLSPKRKLQRALEKHQLCLYYQPIIDIKTEKCI 300
YjcC_33               NHLIFALPAGILGSLVLLLLWLRIRQNYLSPKRKLQRALEKHQLCLYYQPIIDIKTEKCI 300
YjcC_MG1655           NHLIFALPAGILGSLVLLLLWLRIRQNYLSPKRKLQRALEKHQLCLYYQPIIDIKTEKCI 300
YjcC_Fec67            NHLIFALPAGILGSLVLLLLWLRIRQNYLSPKRKLQRALEKHQLCLYYQPIIDIKTEKCI 300
YjcC_Tob1             NHLIFALPAGILGSLVLLLLWLRIRQNYLSPKRKLQRALEKHQLCLYYQPIIDIKTEKCI 300
YjcC_CVM_N17EC1334    NHLIFALPAGILGSLVLLLLWLRIRQNYLSPKRKLQRALEKHQLCLYYQPIIDIKTEKCI 300
YjcC_BLSE174          NHLIFALPAGILGSLVLLLLWLRIRQNYLSPKRKLQRALEKHQLCLYYQPIIDIKTEKCI 300
                      ************************************************************

YjcC_EPECa14          GAEALLRWLGEQGQIMNPAEFIPLAEKEGMIEQITDYVIDNVFRDLGAYLATHADRYVSI 360
YjcC_Fec101           GAEALLRWLGEQGQIMNPAEFIPLAEKEGMIEQITDYVIDNVFRDLGDYLATHADRYVSI 360
YjcC_FSIS11705879     GAEALLRWPGEQGQIMNPAEFIPLAEKEGMIEQITDYVIDNVFRDLGDYLATHADRYVSI 360
YjcC_33               GAEALLRWPGEQGQIMNPAEFIPLAEKEGMIEQITDYVIDNVFRDLGDYLATHADRYVSI 360
YjcC_MG1655           GAEALLRWPGEQGQIMNPAEFIPLAEKEGMIEQITDYVIDNVFRDLGDYLATHADRYVSI 360
YjcC_Fec67            GAEALLRWPGEQGQVMNPAEFIPLAEKEGMIEQVTDYVIDNVFRDLGAYLATHTDRYVSI 360
YjcC_Tob1             GAEALLRWPGEQGQVMNPAEFIPLAEKEGMIEQVTDYVIDNVFRDLGAYLATHTDRYVSI 360
YjcC_CVM_N17EC1334    GAEALLRWLGEQGQIMNPAEFIPLAEKEGMIEQITDYVIDNVFRDLGDYLATHADRYVSI 360
YjcC_BLSE174          GAEALLRWLGEQGQIMNPAEFIPLAEKEGMIEQITDYVIDNVFRDLGAYLATHADRYVSI 360
                      ******** *****:******************:************* *****:******

YjcC_EPECa14          NLSASDFHTSRLIARINQKTEQYAVRPQQIKFEVTEHAFLDVDKMTPIILAFRQAGYEVA 420
YjcC_Fec101           NLSASDFHTSRLIARINQKTEQYAVRPQQIKFEVTEHAFLDVEKMTPIILAFRQAGYEVA 420
YjcC_FSIS11705879     NLSASDFHTSRLIARINQKTEQYAVRPQQIKFEVTEHAFLDVDKMTPIILAFRQAGYEVA 420
YjcC_33               NLSASDFHTSRLIARINQKTEQYAVRPQQIKFEVTEHAFLDVDKMTPIILAFRQAGYEVA 420
YjcC_MG1655           NLSASDFHTSRLIARINQKTEQYAVRPQQIKFEVTEHAFLDVDKMTPIILAFRQAGYEVA 420
YjcC_Fec67            NLSASDFHTSRLIARTNQKTEQHAVRPQQIKFEVTEHAFLDVDKMTPIILAFRKAGYEVA 420
YjcC_Tob1             NLSASDFHTSRLIARTNQKTEQHAVRPQQIKFEVTEHAFLDVDKMTPIILAFRKAGYEVA 420
YjcC_CVM_N17EC1334    NLSASDFHTSRLIARINQKTEQYAVRPQQIKFEVTEHAFLDVEKMTPIILAFRQAGYEVA 420
YjcC_BLSE174          NLSASDFHTSRLIARINQKTEQYAVRPQQIKFEVTEHAFLDVEKMTPIILAFRQAGYEVA 420
                      *************** ******:*******************:**********:******

YjcC_EPECa14          IDDFGIGYSNLHNLKSLNVDILKIDKSFVETLTTHKTSHLIAEHIIELAHSLGLKRSLKA 480
YjcC_Fec101           IDDFGIGYSNLHNLKSLNVDILKIDKSFVETLTTHKTSHLIAEHIIELAHSLGLKTIAEA 480
YjcC_FSIS11705879     IDDFGIGYSNLHNLKSLNVDILKIDKSFVETLTTHKTSHLIAEHIIELAHSLGLKTIAEG 480
YjcC_33               IDDFGIGYSNLHNLKSLNVDILKIDKSFVETLTTHKTSHLIAEHIIELAHSLGLKTIAEG 480
YjcC_MG1655           IDDFGIGYSNLHNLKSLNVDILKIDKSFVETLTTHKTSHLIAEHIIELAHSLGLKTIAEG 480
YjcC_Fec67            IDDFGIGYSNLHNLKSLNVDILKIDKSFVETLTTHKTSHLIAEHIIELAHSLGLKTIAEG 480
YjcC_Tob1             IDDFGIGYSNLHNLKSLNVDILKIDKSFVETLTTHKTSHLIAEHIIELAHSLGLKTIAEG 480
YjcC_CVM_N17EC1334    IDDFGIGYSNLHNLKSLNVDILKIDKSFVETLTTHKTSHLIAEHIIELAHSLGLKTIAEG 480
YjcC_BLSE174          IDDFGIGYSNLHNLKSLNVDILKIDKSFVETLTTHKTSHLIAEHIIELAHSLGLKTIAEG 480
                      *******************************************************   :.

YjcC_EPECa14          SKLRSRLTGCANAACAIARDVFLRRRCRRRCLCNGWSNYPRGS----- 523
YjcC_Fec101           SKLRSRLTGCANAACAIARDGSLRRRCRRRCLCNGWSNYPRGS----- 523
YjcC_FSIS11705879     VV-----TGCANAACAIARDGSLRRRCRRRCLCNGWSNYPRGS----- 518
YjcC_33               VETESRLTGCANAACAIARDGSLRRRCRRRCLCNGWSNYPRGS----- 523
YjcC_MG1655           VETEEQVNWLRKRGVRYCQGWFFAKAMPPQVFMQWMEQLPARELTRGQ 528
YjcC_Fec67            VETEAQVNWLRKHGVRYCQGWFFAKAMPPQVFMQWMG----------- 517
YjcC_Tob1             VETEAQVNWLRKRGVRYCQGWFFAKAMPPQVFMQWMG----------- 517
YjcC_CVM_N17EC1334    VETEEQVNWLRKRGVRYCQGWFFAKAMPPQVFMQWMEQLPARELTRGQ-- 500
YjcC_BLSE174          VETEE------------------------------------------- 485
```

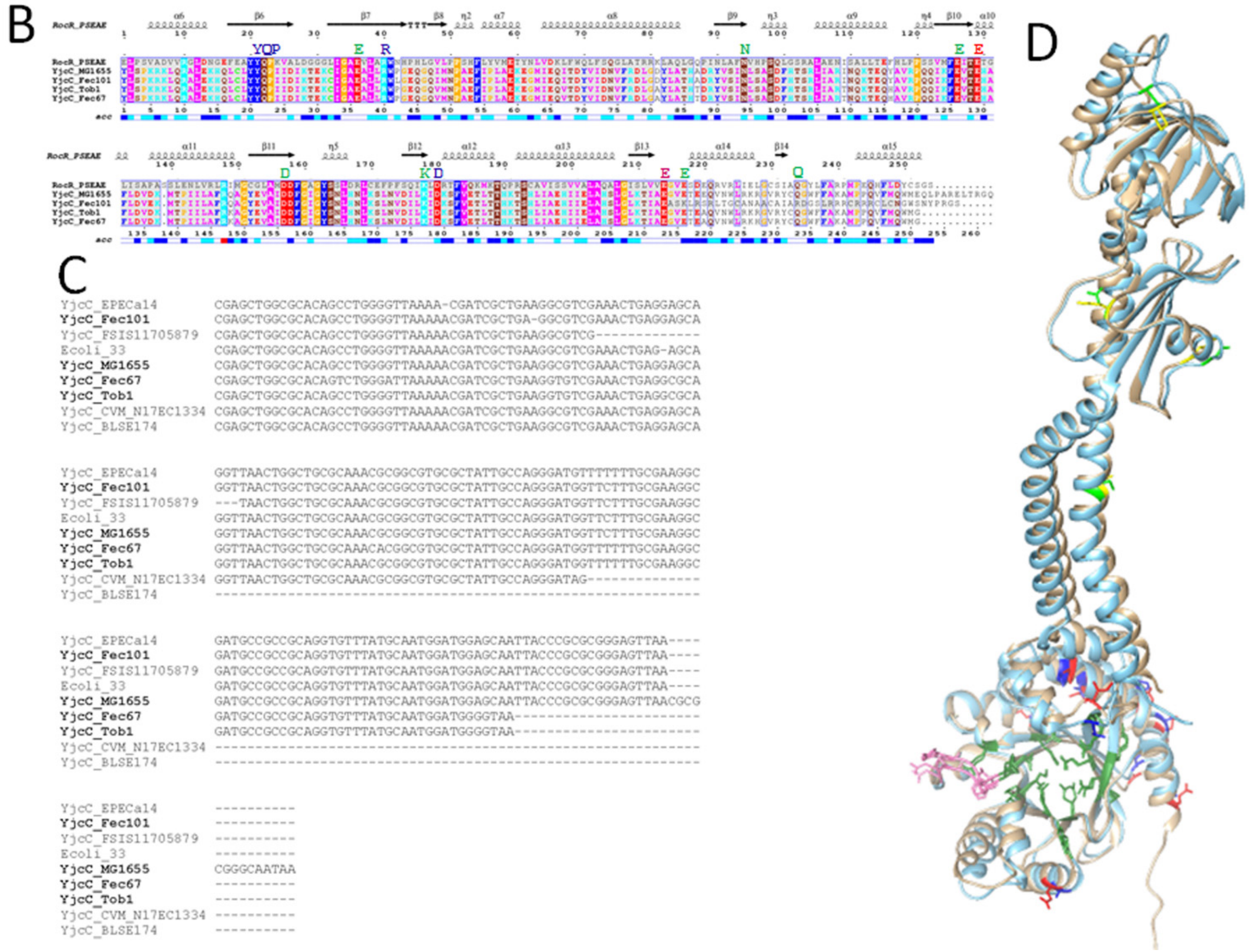

Figure S2. Multiple Alignment of variants of EAL phosphodiesterase YjcC of *E. coli* strains MG1655, Tob1, Fec67 and Fec101. (**A**) Annotated YjcC of investigated *E. coli* strains K-12 MG1655, Tob1, Fec67 and Fec101 [13] and database derived YjcC protein sequences from *E. coli* EPECa14, FSIS11705879. 33, CVM_N17EC1334 and BLSE174 were aligned using Clustal 2.1 [21]. Amino acids deviating from the *E. coli* K-12 MG1655 sequence are displayed in red (for the YjcC variant from Fec101 as investigated in this work) and green (for the YjcC variants from database strains). (**B**) Alignment of EAL domain of YjcC from *E. coli* strains MG1655, Tob1, Fec67 and Fec101 displaying the conservation of consensus amino acid motifs. $YjcC_{Fec101}$ is missing divalent cation binding amino acids at the C-terminal end. (**C**) 3′ end of the open reading frame of *yjcC* showing the nucleotide deletions and substitutions leading to an altered or truncated C-terminus of the gene product. (**D**) Overlaid alpha fold structural models of $YjcC_{MG1655}$ and $YjcC_{Tob1}$ indicating the variant amino acids in blue (green) and red (yellow) for the EAL domain (N-terminal part of YjcC). Catalytic amino acids are indicated in green and loop 6 required for catalytic activity is in magenta.

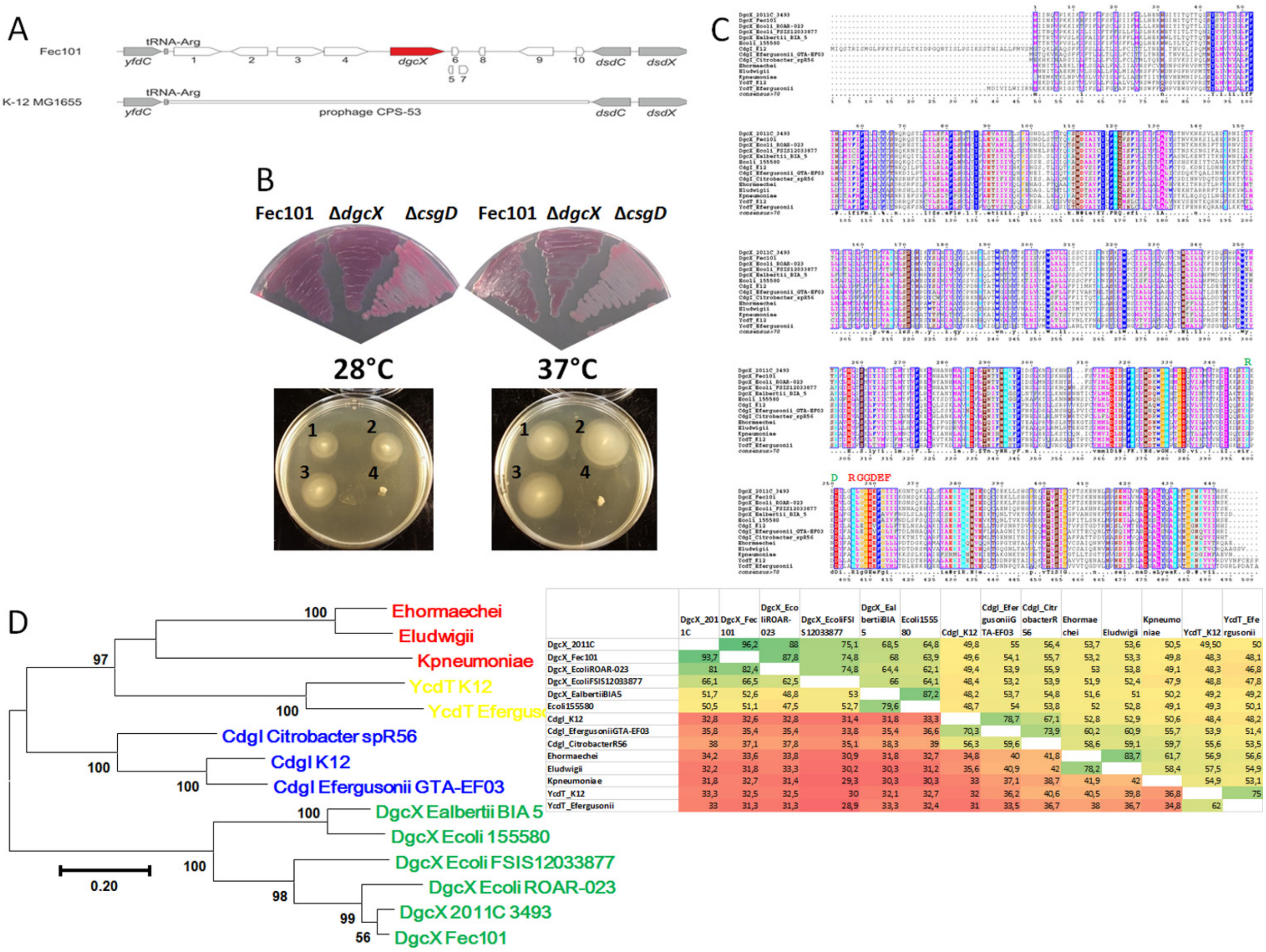

Figure S3. Characterisation of the DGC DgcX of *E. coli* Fec101. (**A**) Flanking region of the DGC encoding gene *dgcX* in Fec101. Genome context for *dgcX* in *E. coli* strain Fec101, modified with reference to annotation by the RAST server. The gene *dgcX* is present on a 9 kbp fragment containing phage-associated genes inserted between the core genome genes *argW* and *dsdC* in the genome of *E. coli* Fec101. *E. coli* K-12 MG1655 harbors the CPS-53 prophage at this position. 1 phage integrase, 2 hypothetical protein, 3 NADH-dependent butanol dehydrogenase, 4 Dienelactone hydrolase, 5 Phage tail fiber assembly protein, 6, 7 Phage exonuclease, 8 putative membrane protein, 9 Transposase, 10 Gene 18 protein. (**B**) Rdar morphotype and motility of the *dgcX* and *csgD* deletion mutant compared to wild type *E. coli* Fec101. To assess rdar morphotype expression, cells were incubated on LB without salt Congo red agar plates for 48 h at 28 °C and for 24 h at 37 °C. To assess motility, cells were inoculated into LB 0.25% agar plates and incubated for 6 h for 28°C and 37°C. 1, Fec101; 2, Fec101 Δ*dgcX*; 3, Fec101 Δ*csgD*; 4, *S.* typhimurium MAE108 (swimming motility negative control. (**C**) Alignment of selected DgcX, CdgI and YcdT variants. Multiple sequence alignment displayed with ESPript [24]. Consensus sequence indicates identity (capital letters), >50% identity (lowercase), ! any of IV; $ any of LM, % any of FY, # any of NDQE. The allosteric cyclic di-GMP binding RxxD is indicated in green and the catalytic GGDEF motif is indicated in red above the alignment. (**D**) Phylogenetic tree of the selected DgcX, CdgI and YcdT variants and heatmap displaying the respective % identity and % similarity.

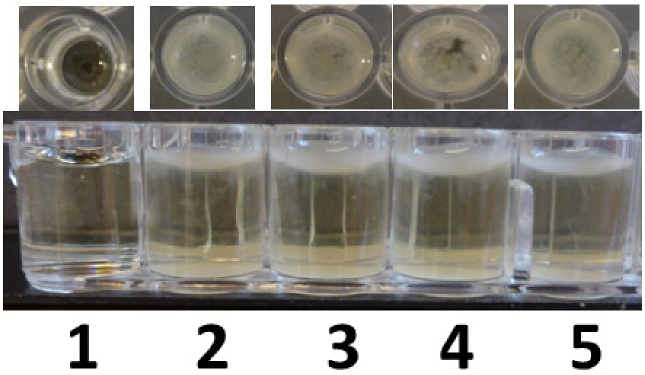

Figure S4. Pellicle assay indicative for the expression of type 1 fimbriae. Bacteria were grown in LB medium in 96 well plates at 37°C for 48 h. Top and side view. 1, medium control; 2, *E. coli* Tob1 (pBad30):; 3, *E. coli* Tob1 (pYcgG$_{Tob1}$); 4, *E. coli* Tob1 (pYcgG$_{B-11870}$); 5, *E. coli* Tob1 (pYcgG$_{MG1655}$). pBAD30=vector control; YcgG variants cloned in pBAD30.

Table S1. Strains constructed or used in this study

Strain	Genotype and Description	Reference
**commensal *E. coli* strains**		
Tob1	rdar$_{28°C}$ /rdar$_{37°C}$, wild type	[1,2]
Fec10	rdar$_{28°C}$ /saw$_{37°C}$	[1,3]
Fec67	rdar$_{28°C}$ /rdar$_{37°C}$	[1]
Fec101	rdar$_{28°C}$ /rdar$_{37°C}$	[1]
**uropathogenic *E. coli* strains**		
B-8638	rdar$_{28°C}$ /rdar$_{37°C}$	[4]
B-11870	rdar$_{28°C}$ /rdar$_{37°C}$	[2]
No. 12	rdar$_{28°C}$ /rdar$_{37°C}$	[5]
**reference strains**		
E. coli K-12 MG1655	F- lambda- *ilvG*- *rfb*-50 *rph*-1	Laboratory collection
Salmonella typhimurium MAE108	MAE52 (UMR1 (ATCC 14028 Nal^r)) Δ*csgD fliC5050::*MudJ *fljB5001::*MudCm	[6]
***E. coli* strain mutants**		
Fec101_d*csgD*	Fec101 Δ*dgcX::Km*	This work
Tob2	Tob1 Δ*csgD*	[1]
Fec10_d*csgD*	Fec10 Δ*csgD*	[2]
Pseudomonas aeruginosa		
Pseudomonas aeruginosa SG17M	*P. aeruginosa* SG17M pPN105::ClpG-6xHis	[7]

Table S2. Plasmids constructed or used in this study

**Plasmid cloned sequence**	**Plasmid name**	**Description**	**Reference**
empty vector	pBAD30	cloning vector, pACYC origin, arabinose inducible pBAD promoter, Amp^r	[8]
kanamycin resistance cassette	pKD4	template plasmid for λ red mediated recombination, Km^r, Amp^r	[9]
λ red recombinase	pKD46	pBAD promoter, encodes λ red recombinase, Amp^r	[9]
pBAD30::$YcgG_{MG1655}$	pYcgG1	Amp^r, C-terminal 6xHis	This study
pBAD30::$YcgG_{Tob1}$	pYcgG2	Amp^r, C-terminal 6xHis	This study
pBAD30::$YcgG_{B\text{-}11870}$	pYcgG3	Amp^r, C-terminal 6xHis	This study
pBAD30::$DgcX_N$	pDgcX1	Amp^r, N-terminal 6xHis	This study
pBAD30::$DgcX_C$	pDgcX2	Amp^r, C-terminal 6xHis	This study
pBAD30::$DgcX_{E359A}$	pDgcX3	Amp^r, N-terminal 6xHis, GGAEF mutation	This study
pBAD30::$DgcX_{E360A}$	pDgcX4	Amp^r, N-terminal 6xHis, GGEAF mutation	This study
pBAD30::$DgcX_{E359A/unE360A}$	pDgcX5	Amp^r, N-terminal 6xHis, GGAAF mutation	This study
pBAD30::$YjcC_{MG1655}$	pYjcC1	Amp^r	This study
pBAD30::$YjcC_{Tob1}$	pYjcC2	Amp^r	This study
pBAD30: $YjcC_{Fec67}$	pYjcC3	Amp^r	This study
pBAD30::$YjcC_{Fec101}$	pYjcC4	Amp^r	This study
pBAD30::$YjcC_{Tob1}$	pYjcC2	Amp^r	This study

Table S3. Oligonucleotides used in this study

**Name**	**Primer sequence**	**Purpose**
YcgG_Fec101MGfw	GCG *TCTAGA* TAAGGAGGTTTTATCATGCGCAATACACT-CATACC	Cloning YcgG from *E. coli* MG1655
YcgG_Tob1fw	GCG *TCTAGA* TAAGGAGGTTTTATC ATGGAGAGCGCTCGT-TTTCC	Cloning YcgG from *E. coli* Tob1
YcgG_6xHis_rv	GCG *AAGCTT* TCA **GTGATGATGATGATGATG** CTCAACC-ACAACCTTCACCT	Cloning YcgG from *E. coli* Tob1 and MG1655
YcgG_11870fw	GCG *TCTAGA* TAAGGAGGTTTTATCTTGATATTTATTCTGT-TAAT	Cloning YcgG from *E. coli*-B-11870
YcgG_6His11870rv	GCG *AAGCTT* TCA **GTGATGATGATGATGATG** CTCAAC-CACAATCTTCACCT	Cloning YcgG from *E. coli* B-11870
YjcC_fw	TTAA *GGTACC* AGGAGGTAATATATGAGTCATCGTGCA-CGACA	Cloning YjcC from *E. coli* MG1655, Tob1, Fec67 and Fec101
YjcC_rv	AGTT *TCTAGA*_TTGATCGCACTCCCAGCG	Cloning YjcC from *E. coli* MG1655, Tob1, Fec67 and Fec101
dgcX-Nhis-pBAD_Start	CTAG T*TCTAGA* ATGCACCATCACCACCATCATATTAT-CAATAAAGTACCCAAGAA	Cloning of *dgcX* from *E. coli* Fec101 into pBAD30 with an N-terminal 6xHis-tag
dgcX-Nhis-pBAD_stop	AATTGA *AAGCTT* TTATTTTGAGTGGATTATTACCTTG	Cloning of *dgcX* from *E. coli* Fec101 into pBAD30 with an N-terminal 6xHis-tag
dgcX-Chis-pBAD_Start	CTAGT *TCTAGA* ATGATTATCAATAAAGTACCCAAGAA	Cloning of *dgcX* from Fec101 into pBAD30 with a C-terminal 6xHis-tag
dgcX-Chis-pBAD_Stop	AATTGA *AAGCTT* TTAATGATGGTGGTGATGGTGTTTTGA-GTGGATTATTACCTTG	Cloning of *dgcX* from Fec101 into pBAD30 with a C-terminal 6xHis-tag
DgcX-Mut-GGAEF	CGCTTAGGCGGTG***CGG***AGTTCGGTATTATCATAAAAGG	Forward primer for site directed mutagenesis of *dgcX* from *E. coli* FEC101 changing GGEEF to GGAEF
DgcX-Mut-GGEAF	CGCTTAGGCGGTGAAG***CGT***TCGGTATTATCATAAAAGG	Forward primer for site directed mutagenesis of *dgcX* from *E. coli* FEC101 changing GGEEF to GGEAF
DgcX-Mut-GGAAF	CGCTTAGGCGGTG***CGGCGT***TCGGTATTATCATAAAAGG	Forward primer for site directed mutagenesis of *dgcX* from *E. coli* FEC101 changing GGEEF to GGAAF
Mut-DgcX-GGEEF-Rev	CACCGCCTAAGCGCCC	Reverse primer for site directed mutagenesis of the GGEEF motif of *dgcX* from *E. coli* FEC101
GGAEF-control	GCGCTTAGGCGGTGCG	Primer used to confirm mutation of GGEEF to GGAEF
GGEAF-control	CGCTTAGGCGGTGAAGC	Primer used to confirm mutation of GGEEF to GGEAF
GGAAF-control	GCTTAGGCGGTGCGGC	Primer used to confirm mutation of GGEEF to GGAAF
dgcX-KOfor1	CAATACTCGCTGAATCGTCATTTGTCGACGTGGAATATCG GTGTAGGCTGGAGCTGCTTCG	Deletion of *dgcX* from strain *E. coli* Fec101
dgcX-KOrev	CCCGGTTCTTTTGGCTTGATATAACGCTTTATCGGCATTGA CCATATGAATATCCTCCTTAG	Deletion of *dgcX* from strain *E. coli* Fec101

DgcX-ctrl-fw	GCCTGACAACCTTATCCTACC	Control primer for Δ*dgcX* from *E. coli* Fec101
DgcX-ctrl-rv	GATATTGAGT GGCCAACAGTACCC	Control primer for Δ*dgcX* from *E. coli* Fec101

In italics, restriction site; **in bold**, 6xHis-tag coding sequence; <u>underlined</u>, pKD4 sequence; ***bold and italics***, mutated nucleotides in site directed mutagenesis.

References:

1. Bokranz, W.; Wang, X.; Tschäpe, H.; Römling, U. Expression of cellulose and curli fimbriae by *Escherichia coli* isolated from the gastrointestinal tract. *J Med Microbiol* **2005**, *54*, 1171-1182.
2. Cimdins, A.; Simm, R.; Li, F.; Lüthje, P.; Thorell, K.; Sjöling, A.; Brauner, A.; Römling, U. Alterations of c-di-GMP turnover proteins modulate semi-constitutive rdar biofilm formation in commensal and uropathogenic *Escherichia coli*. *Microbiologyopen* **2017**, *6*.
3. Kamal, S.M.; Cimdins-Ahne, A.; Lee, C.; Li, F.; Martin-Rodriguez, A.J.; Seferbekova, Z.; Afasizhev, R.; Wami, H.T.; Katikaridis, P.; Meins, L.; et al. A recently isolated human commensal *Escherichia coli* ST10 clone member mediates enhanced thermotolerance and tetrathionate respiration on a P1 phage-derived IncY plasmid. *Mol Microbiol* **2021**, *115*, 255-271.
4. Cimdins, A.; Lüthje, P.; Li, F.; Ahmad, I.; Brauner, A.; Römling, U. Draft genome sequences of semiconstitutive red, dry, and rough biofilm-forming commensal and uropathogenic *Escherichia coli* isolates. *Genome Announc* **2017**, *5*.
5. Kai-Larsen, Y.; Lüthje, P.; Chromek, M.; Peters, V.; Wang, X.; Holm, A.; Kadas, L.; Hedlund, K.O.; Johansson, J.; Chapman, M.R.; et al. Uropathogenic *Escherichia coli* modulates immune responses and its curli fimbriae interact with the antimicrobial peptide LL-37. *PLoS Pathog* **2010**, *6*, e1001010.
6. Rochon, M.; Römling, U. Flagellin in combination with curli fimbriae elicits an immune response in the gastrointestinal epithelial cell line HT-29. *Microbes Infect* **2006**, *8*, 2027-2033.
7. Lee, C.; Franke, K.B.; Kamal, S.M.; Kim, H.; Lünsdorf, H,; Jäger, J.; Nimtz, M.; Trček, J.; Jänsch, L.; Bukau, B.; et al. Stand-alone ClpG disaggregase confers superior heat tolerance to bacteria. *Proc Natl Acad Sci U S A* **2018,** 115, E273-E282.
8. Guzman, L.M.; Belin, D.; Carson, M.J.; Beckwith, J. Tight regulation, modulation, and high-level expression by vectors containing the arabinose PBAD promoter. *J Bacteriol* **1995**, *177*, 4121-4130.
9. Datsenko, K.A.; Wanner, B.L. One-step inactivation of chromosomal genes in *Escherichia coli* K-12 using PCR products. *Proc Natl Acad Sci U S A* **2000**, 97, 6640-6645.
